# A genomic snapshot of demographic and cultural dynamism in Upper Mesopotamia during the Neolithic Transition

**DOI:** 10.1126/sciadv.abo3609

**Published:** 2022-11-04

**Authors:** N. Ezgi Altınışık, Duygu Deniz Kazancı, Ayça Aydoğan, Hasan Can Gemici, Ömür Dilek Erdal, Savaş Sarıaltun, Kıvılcım Başak Vural, Dilek Koptekin, Kanat Gürün, Ekin Sağlıcan, Daniel Fernandes, Gökhan Çakan, Meliha Melis Koruyucu, Vendela Kempe Lagerholm, Cansu Karamurat, Mustafa Özkan, Gülşah Merve Kılınç, Arda Sevkar, Elif Sürer, Anders Götherström, Çiğdem Atakuman, Yılmaz Selim Erdal, Füsun Özer, Aslı Erim Özdoğan, Mehmet Somel

**Affiliations:** ^1^ Human-G Laboratory, Department of Anthropology, Hacettepe University, 06800 Beytepe, Ankara, Turkey.; ^2^ Department of Biological Sciences, Middle East Technical University, 06800 Ankara, Turkey.; ^3^ Department of Settlement Archaeology, Middle East Technical University, 06800 Ankara, Turkey.; ^4^ Husbio-L Laboratory, Department of Anthropology, Hacettepe University, 06800 Beytepe, Ankara, Turkey.; ^5^ Department of Museology and Cultural Heritage Management, Çanakkale Onsekiz Mart University, Çanakkale 17100, Turkey.; ^6^ Department of Health Informatics, Graduate School of Informatics, Middle East Technical University, 06800 Ankara, Turkey.; ^7^ Department of Evolutionary Anthropology, University of Vienna, Vienna, Austria.; ^8^ Human Evolution and Archaeological Sciences, University of Vienna, Vienna, Austria.; ^9^ CIAS, Department of Life Sciences, University of Coimbra, Coimbra, Portugal.; ^10^ Centre for Palaeogenetics, Stockholm, Sweden.; ^11^ Department of Archaeology and Classical Studies, Stockholm University, Stockholm, Sweden.; ^12^ Department of Bioinformatics, Graduate School of Health Sciences, Hacettepe University, 06100 Sıhhiye, Ankara, Turkey.; ^13^ Department of Modeling and Simulation, Graduate School of Informatics, Middle East Technical University, 06800 Ankara, Turkey.; ^14^ Department of Archaeology, Çanakkale Onsekiz Mart University, Çanakkale 17100, Turkey.

## Abstract

Upper Mesopotamia played a key role in the Neolithic Transition in Southwest Asia through marked innovations in symbolism, technology, and diet. We present 13 ancient genomes (c. 8500 to 7500 cal BCE) from Pre-Pottery Neolithic Çayönü in the Tigris basin together with bioarchaeological and material culture data. Our findings reveal that Çayönü was a genetically diverse population, carrying mixed ancestry from western and eastern Fertile Crescent, and that the community received immigrants. Our results further suggest that the community was organized along biological family lines. We document bodily interventions such as head shaping and cauterization among the individuals examined, reflecting Çayönü’s cultural ingenuity. Last, we identify Upper Mesopotamia as the likely source of eastern gene flow into Neolithic Anatolia, in line with material culture evidence. We hypothesize that Upper Mesopotamia’s cultural dynamism during the Neolithic Transition was the product not only of its fertile lands but also of its interregional demographic connections.

## Teaser

Neolithic Upper Mesopotamia was a center of cultural innovation; ancient genomes now reveal that it was also a migration hub.

## INTRODUCTION

**Fig. 1.**
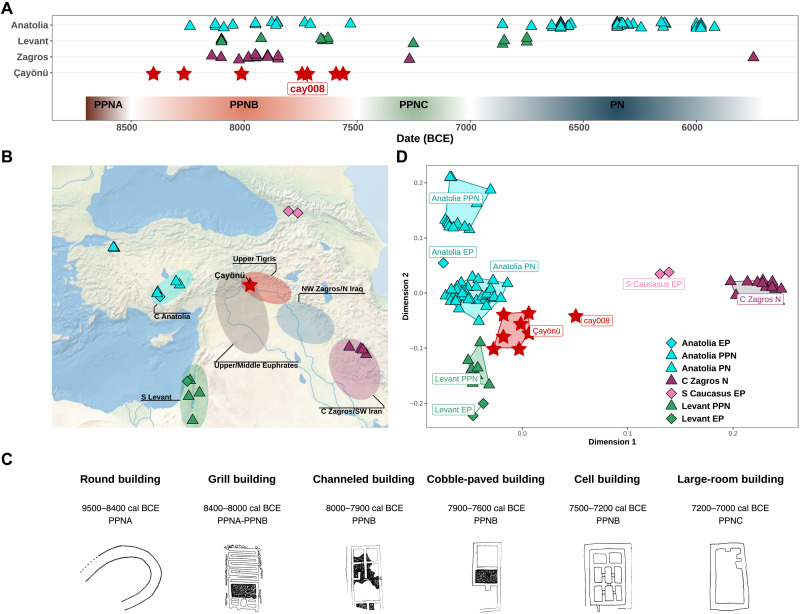
Spatiotemporal distribution of the samples and the population structure of Neolithic Southwest Asia. (**A**) Timeline of ancient Southwest Asian individuals used in the analyses. Colored horizontal bars at the bottom represent the subperiods of the Neolithic Era in Southwest Asia. (**B**) The map shows EP and Neolithic populations from Southwest Asia. Shaded areas mark PPN period cultural zones (referred to as the Aceramic period in C Anatolia). (**C**) Çayönü building types and their approximate dates of use, considered as evidence for Çayönü’s cultural openness and ingenuity. Modified from (*112*). (**D**) The first two dimensions of the MDS plot of genetic distances. The MDS summarizes the genetic distance matrix among ancient genomes calculated as (1 − outgroup *f*_3_) values. Outgroup *f*_3_-statistics were calculated as *f*_3_(*Yoruba; individual_1_, individual_2_*). The labels represent the following sites: Anatolia EP: Pınarbaşı; Anatolia PPN: Boncuklu and Aşıklı Höyük; Anatolia PN: Çatalhöyük and Barcın Höyük; Levant EP: Natufian; Levant PPN: Ain’ Ghazal, Kfar HaHoresh, Motza, and Ba’ja; C Zagros N (Central Zagros Neolithic): Ganj Dareh, Tepe Abdul, and Wezmeh Cave; S Caucasus EP (South Caucasus EP): Kotias and Satsurblia. See note S5 for a definition of “Anatolia.” PPNA, Pre-pottery Neolithic A; PPNB, Pre-pottery Neolithic B; PPNC, Pre-pottery Neolithic C.

Located between the Euphrates and Tigris rivers, the hilly flanks of Upper Mesopotamia were home to the earliest sedentary hunter-gatherers who built the first monumental structures at Göbekli Tepe (*1*) and domesticated numerous local plant and animal species, including einkorn, emmer, sheep, goat, pig, and cattle (*2*–*6*). The innovative spirit and cultural dynamism of these societies during the Neolithic Transition in Southwest Asia (c. 9800 to 6500 BCE) are well documented in the archaeological record, but their demographic history and biological kinship-related traditions have remained unknown owing to the lack of genomes from Upper Mesopotamia. This stands in contrast with a notable number of recent archaeogenomic studies that focused on the three most distant corners of Neolithic Southwest Asia, namely, South Levant, Central Zagros, and Central Anatolia (Fig. 1, A and B) (*7*–*13*). This body of work has together revealed (i) genetically distinct populations across the three regions; (ii) a dominant trend of population continuity between pre-Neolithic, Pre-Pottery Neolithic (PPN), and Pottery Neolithic (PN) communities; and (iii) an overlay of interregional gene flow through time, such as inferred “southern” and “eastern” gene flow events into Central Anatolia between the Early and Late Neolithic (note S1). Meanwhile, key questions about the possible roles of Upper Mesopotamia in interregional demographic and cultural change, e.g., whether Upper Mesopotamia influenced Late Neolithic Central Anatolia and whether it was the source of the post-Neolithic gene flow into Anatolia (*7*, *14*), have remained open. With the exception of a single ancient DNA (aDNA) study reporting 15 mitochondrial DNA sequences from the Upper Euphrates (*15*), Neolithic Upper Mesopotamia has remained genomically unexplored, mostly owing to low DNA preservation in the region.

Here, we address this gap by studying genomic data from Çayönü Tepesi (hereon Çayönü) of the Upper Tigris area (Fig. 1, A and B), a settlement that presents one of the best examples of the transition from foraging to food production in Southwest Asia (*16*). First, Çayönü’s uninterrupted stratigraphy extending from the Pre-Pottery Neolithic A (PPNA) (c. 9500 cal BCE) to the final PPN (c. 7000 cal BCE) is unparalleled in the region. Second, the Çayönü Neolithic community is recognized for its marked cultural dynamism, which is reflected (i) in evidence for continuous plant management and cultivation (*17*) and animal management (pig, cattle, sheep, and goat) (*18*), (ii) in continuous innovation in architectural styles (Fig. 1C), and (iii) in technological experimentation, such as pioneering lime burning techniques (*16*, *19*) and the production of copper and malachite artifacts, including beads, inlays, etc. (*20*). Last, both western (Levant-Euphrates) and eastern (Tigris-Zagros) influences and parallel developments are traceable in Çayönü’s material culture (table S2 and note S2) (*21*). These observations suggest that Çayönü and contemporaneous Upper Mesopotamian communities could have acted as hubs of cultural interaction and innovation in Neolithic Southwest Asia.

Our study presents genomic data from Çayönü, which we then use to describe (i) the demographic makeup of the Fertile Crescent populations and its relation to material culture affinities observed in the archaeological record, (ii) the Neolithic demographic transition as reflected in genomic diversity at Çayönü and other Neolithic sites, (iii) genetic kinship among coburials in domestic structures at Çayönü, and (iv) the potential contribution of Upper Mesopotamia to Neolithic and post-Neolithic population movements in Anatolia. We also detail the curious case of an infant burial at Çayönü, whom we infer to be a migrant offspring and who presents not only artificial cranial deformation but also one of the earliest known cases of cauterization in her skull.

## RESULTS AND DISCUSSION

We studied the skeletal remains of 33 individuals from Çayönü (Fig. 1, A and B, and table S3). These were mainly found as subfloor burials located inside or within the proximity of six Pre-Pottery Neolithic B (PPNB) buildings (table S1). We screened 33 aDNA libraries by shotgun sequencing, which revealed endogenous DNA proportions varying between 0.04 and 5% (median = 0.2%; fig. S1). This was lower than aDNA preservation in a contemporaneous Central Anatolian settlement, Aşıklı (median = 1.4%, Wilcoxon rank sum test, *P* < 0.05), but comparable to another Central Anatolian site of a similar date, Boncuklu (median = 0.1%, Wilcoxon rank sum test, *P* > 0.05; fig. S2).

Libraries from 14 individuals were chosen for deeper sequencing (Materials and Methods), from which we generated shotgun genomes with depths ranging from 0.016× to 0.49×. (fig. S1 and table S3). High rates of postmortem damage accumulation at read ends, short average fragment sizes [49 to 60 base pairs (bp), median = 51.4 bp], and mitochondrial haplotype-based estimates suggested the authenticity of all 14 libraries (Materials and Methods) (table S3). With these data, we first estimated genetic kinship among all individual pairs (Materials and Methods). Two samples, both identified as female infants (cay018 and cay020), were genetically inferred either to belong to the same individual or to be identical twins. Skeletal analyses also suggested that both petrous bones could belong to the same individual. We therefore merged their genomic data and treated these merged data as representing a single individual, reducing our sample size to 13 (6 adult females, 2 adult males, 3 subadult females, and 2 subadult males). We further identified four related pairs of first to third degree (see below) and removed all but one individual among sets of closely related individuals in population genetic analyses (Materials and Methods).

### The east-west genetic structure of Neolithic Southwest Asia

**Fig. 2.**
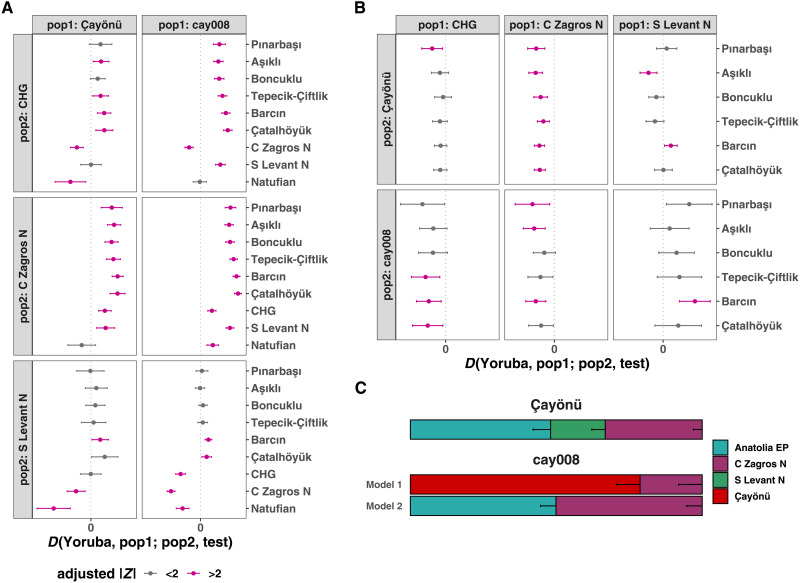
Genetic affinities of the Çayönü population with the neighboring populations. Formal tests computed in the form of (**A**) *D*(*Yoruba, Çayönü/cay008; pop2, test*) and (**B**) *D*(*Yoruba, pop1; Çayönü/cay008, Anatolia EP/PPN/PN*). *Z* scores were corrected with the Benjamini-Hochberg multiple testing correction (*86*). Horizontal bars represent ±2 SE. (**C**) qpAdm modeling of the Çayönü group and cay008. The local Çayönü group or an outlier cay008 individual was the “target”; Central Anatolia EP, Central Zagros Neolithic, and South Levant Neolithic samples were sources for both targets. The local Çayönü group was also used as the “source” for modeling of cay008. Model 1 represents the model with “local Çayönü population + C Zagros N,” whereas model 2 includes “Anatolia EP + C Zagros N” as sources. Horizontal bars represent SEs of the coefficients. All three models yielded *P* > 0.05. We also cannot reject a three-way model of Central Anatolia PPN, Central Zagros, and South Levant at *P* > 0.01, while three-way models for cay008 were not infeasible (table S5). In all analyses shown in the figure, “Çayönü” represents the nine genomes listed in Table 1, excluding relatives and cay008.

To obtain an overview of genetic affinities among human populations in Neolithic Southwest Asia, we compared the 13 Çayönü genomes with published ancient genomes dating to c. 15,000 to 5500 BCE from the Fertile Crescent and neighboring regions (table S4) (*7*–*9*, *12*, *13*, *22*–*25*) using multidimensional scaling (MDS) of pairwise *f*_3_ results, *D*-statistics, and qpAdm analyses (*26*). These led to a number of observations. In the MDS analysis, the Çayönü group occupied a distinct and intermediate position within the space of Southwest Asian genetic diversity bordered by early Holocene South Levant, Central Zagros and South Caucasus, and Central Anatolia (Fig. 1D and fig. S3). Our sample of Çayönü genomes was internally homogeneous within this space, with the exception of an “outlier” individual, cay008, who appeared relatively closer to Zagros/Caucasus individuals. *D*-statistics likewise showed that the Çayönü group was genetically closer to western Southwest Asia, and particularly to early Holocene Central Anatolia, than to eastern Southwest Asia (Central Zagros) (Fig. 2A and table S5). At the same time, Central Zagros genomes showed higher genetic affinity to our Çayönü sample than to Central Anatolia or South Levant (Fig. 2B). Last, we found that cay008 harbors higher Zagros contribution than other Çayönü individuals (Figs. 1D and 2C, figs. S4 to S6, and table S6).

**Fig. 3.**
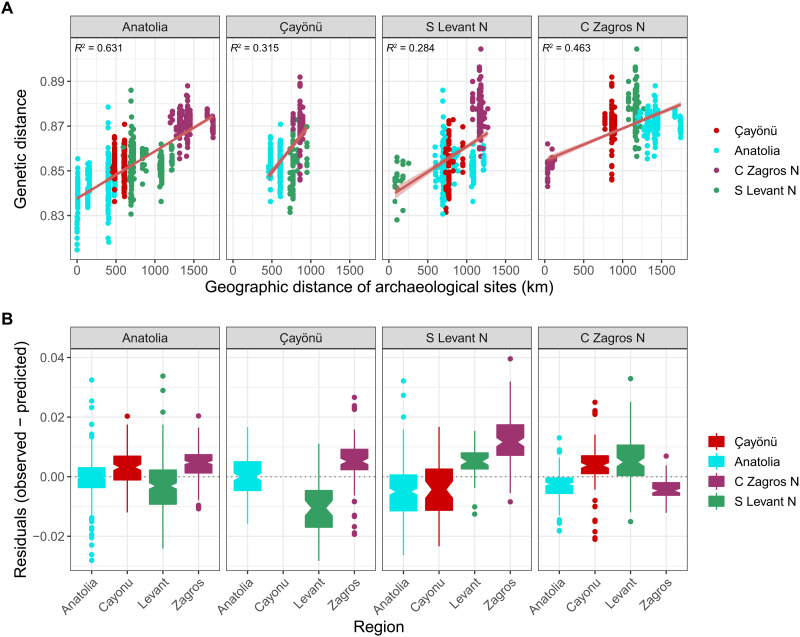
Genetic isolation by distance in Southwest Asia. (**A**) Correlation between geographic (*x* axis) and genetic (*y* axis) distance for Southwest Asia Neolithic populations. The red regression line shows the linear fit with 95% confidence interval. Each point represents pairs of individuals from Southwest Asia Neolithic. Pairs from the same site and pairs separated by >1000 years of time difference were not included. All regression lines were highly significant (*P* < 0.001). (**B**) The distribution of residuals that we calculated by subtracting the observed values from the predicted values obtained from the linear regression models in (A). In all analyses shown in the figure, Çayönü represents the nine genomes listed in Table 1, excluding relatives and cay008.

Given these observations, we first investigated the origins of the genetic structure in Neolithic Southwest Asia. The higher genetic affinity among Upper Mesopotamia (represented by Çayönü), Central Anatolia, and South Levant populations relative to Central Zagros (Fig. 1D and fig. S3) was intriguing, which led us to ask whether this affinity could be explained by an isolation-by-distance process (*27*, *28*). We computed shared genetic drift between each pair of individuals in our Southwest Asia sample and compared these to geodesic geographic distance among settlements (Materials and Methods). To eliminate the effect of temporal genetic changes, we only included individual pairs separated by <1000 years. We found a general correlation between spatial and genetic distances, as expected (Fig. 3A). However, we also found that Central Zagros genomes were significantly more differentiated compared to that expected from a linear isolation-by-distance model (Fig. 3B). We therefore infer a clearly differentiated east-west genetic structure in the Fertile Crescent, where the lowest effective migration (*29*) appears to lie between Upper Mesopotamia and the Central Zagros (note S3).

At face value, this result may seem to imply resistance to gene flow between Upper Mesopotamia and Central Zagros during the Neolithic. However, this is probably not a valid explanation, as such resistance does not align with genetic evidence for interregional migration (presented by Zagros-related admixture in Çayönü and also the cay008 individual; see below) and with observed material culture affinities between the two regions during the Neolithic [e.g., (*30*); table S2]. We therefore suggest an alternative scenario to explain the observed genetic structure. During the Last Glacial Maximum, the ancestors of populations inhabiting the eastern regions of Southwest Asia during the early Holocene (the ancestors of our Central Zagros/South Caucasus genomes) could have been partly isolated from the ancestors of populations from western regions (the ancestors of Central Anatolia/Levant genomes). These “east” and “west” populations could have differentiated through drift or by admixture with third populations. Such a scenario also appears in line with archaeological data indicating close interaction within the Zagros sphere (between NW Zagros and Central Zagros populations) and also between Levant and Central Anatolia in the late Paleolithic and Epipaleolithic (EP) (*31*, *32*). It is plausible that east-west admixture occurred in Upper Mesopotamia, giving rise to Çayönü’s gene pool, and may have also influenced Central Anatolia by the PN (*7*). We note that the duration and timing of both the hypothesized isolation and admixture processes remain unclear [e.g., Marchi *et al.* (*33*) estimate an Anatolia-Zagros split in the EP] and that alternative scenarios could also explain the data (e.g., the Upper Mesopotamian gene pool being the product of an east-west cline with variable rates of migration). Irrespective of the demographic mechanisms, though, Central Zagros appears to have been genetically the most distinct group in early Holocene Southwest Asia.

### Admixed ancestry and diverse material culture affinities in Çayönü

We next investigated the demographic origins of Çayönü inhabitants. The *D*-statistics results mentioned above had suggested that the Çayönü sample carried mixed eastern and western ancestry (Fig. 2, A and B), which is consistent with the site’s intermediate geographic position. Using qpAdm, we could further model ancestry proportions in the Çayönü genome sample (excluding the cay008 individual) as three-way admixtures of Central Anatolia (represented by EP Pınarbaşı; Anatolia EP)–, South Levant–, and Central Zagros–related ancestries (Fig. 2C, fig. S4, and table S5) (Materials and Methods). Çayönü bears mainly Anatolian ancestry, complemented by 33% (SE ± 3%) of Zagros and 19% (SE ± 5%) of Southern Levant ancestries (*P* > 0.05, indicating feasibility of the model).

**Fig. 4.**
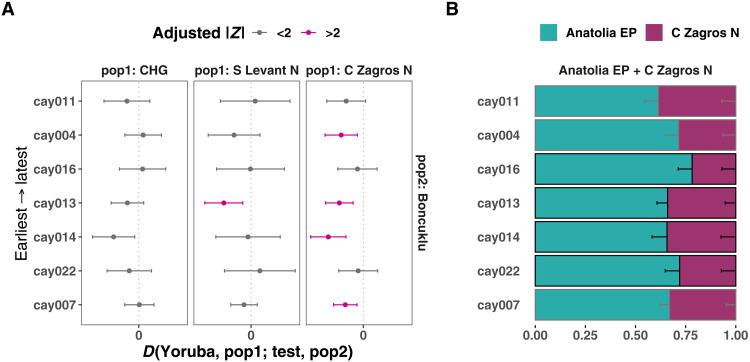
Testing temporal genetic change in Çayönü. (**A**) Formal tests computed in the form of *D*(*Yoruba, pop1; test, Boncuklu*) where *pop1* denotes CHG/S Levant N/C Zagros N, and *test* denotes radiocarbon-dated Çayönü individuals (Table 1), ordered from earliest to latest in bottom to top direction, respectively. Horizontal bars represent ±2 SE. (**B**) qpAdm model of each radiocarbon-dated Çayönü individual. The outlier individual cay008 was excluded. The *y*-axis is in the same order as in (A). Black outlines around the boxes show that the *P* value of the model is >0.05 (indicating feasibility), whereas gray outlines indicate 0.05 > *P* > 0.01.

We then asked whether the genetic affinity of Çayönü individuals to regional populations could have changed over the 1000 years covered by our sample. We found no significant temporal effect (multiple testing corrected *P* > 0.05; Fig. 4A). Testing temporal shifts in qpAdm-based ancestry components across individuals also did not reveal a significant change (*P* > 0.05; Fig. 4B and fig. S7, A to D). Still, these results do not rule out immigration into Çayönü during the PPN, as the cay008 outlier individual exemplifies. With qpAdm, we estimated that the cay008 genome carried 50% Anatolia EP and 50% Central Zagros N ancestry (SE ± 5%, *P* > 0.05) and lacked a significant South Levant component found in the rest of Çayönü genomes (Fig. 2C and fig. S4). We were also able to model cay008 as a mixture of the “local” Çayönü sample (79%, SE ± 8%) and Zagros-like (21%, SE ± 8%) ancestries (*P* > 0.05). Here, we caution that regions other than the Zagros, but with genetically related populations, including the South Caucasus (table S5) or yet unsampled regions, could also be the source of eastern ancestry in Çayönü. In addition, “Zagros-related ancestry” itself might actually represent human mobility from Northwest Zagros (i.e., modern-day North Iraq, from where archaeogenomic data are not yet available) rather than from Central Zagros, which would also be compatible with Çayönü’s material cultural affinities with Northwest Zagros (Fig. 1B and table S2).

These observations overall support the notion that the Çayönü population had both historical and ongoing demographic connections with neighboring regions. Archaeologically, Çayönü shares a number of distinctive features with PPNA/PPNB settlements in the eastern wing of Neolithic Southwest Asia, particularly those in the Tigris and Euphrates basins and Northwest Zagros (Fig. 1B and table S2). These features include monumental architecture and/or special buildings, lithic types such as the “Çayönü tool,” and plain or winged marble bracelets (table S2) (*15*, *21*, *34*, *35*). Another observation worth mentioning is the joint presence of both the pressure technique and bidirectional blade technologies at Çayönü, which were predominant in the eastern and western regions of Neolithic Southwest Asia, respectively (table S2). Obsidian network analyses similarly suggest close interactions between the Tigris and Zagros areas (*30*). We speculate that Çayönü’s east-west mixed ancestry and its possible openness to interregional human movement may have facilitated its observed wide-ranging material culture affinities and cultural dynamism.

### An early Neolithic demographic shift in the “Fertile Crescent”

Our dataset further allowed us to revisit a previous observation on the demographic impact of the Neolithic Transition. It had been earlier reported that the Central Anatolian PPN populations Aşıklı and Boncuklu had low levels of genetic diversity, similar to Upper Paleolithic and Mesolithic Europeans and Caucasians (*9*, *13*). In comparison, Central Anatolian PN populations Tepecik-Çiftlik and Çatalhöyük, as well as West Anatolian and European Neolithic populations, carried higher genetic diversity levels. This temporal increase in genetic diversity was attributed to the transition to farming and associated intensification of population movements and admixture [(*9*); also see (*12*)].

**Fig. 5.**
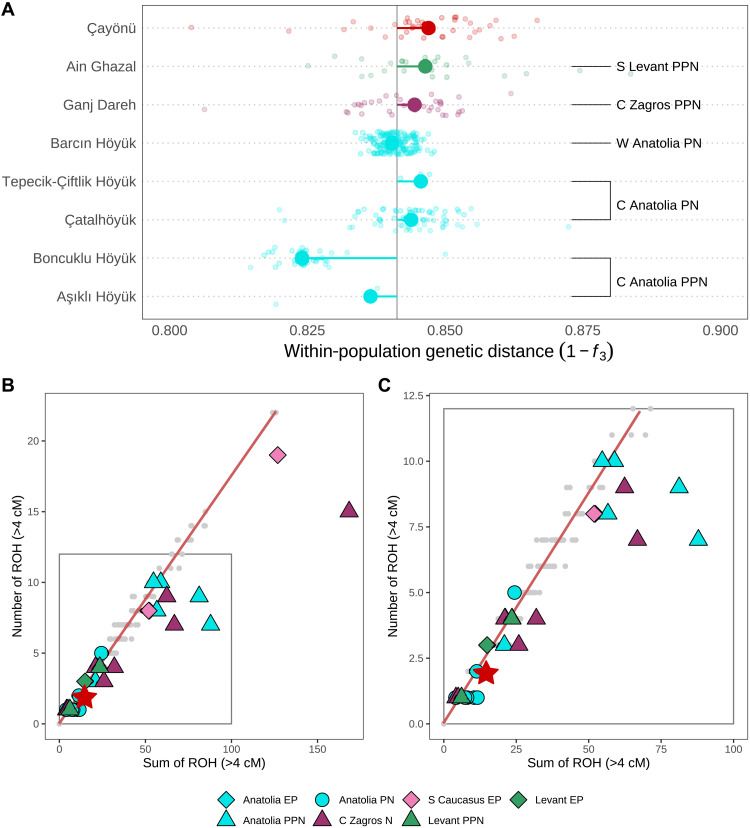
Genetic diversity in Neolithic Southwest Asia. (**A**) Small dots show pairwise genetic distance calculated as (1 − outgroup *f*_3_) values for all pairs of individuals, whereas large dots show the median values of each population. The vertical gray line represents the total median across the eight populations. Deviation from the total median is shown with colored horizontal lines. Outgroup *f*_3_-statistics were computed as *f*_3_(*Yoruba; ind_1_, ind_2_*) where *ind_1_* and *ind_2_* represent individuals from the same archaeological site. (**B**) and (**C**) present ROHs in Southwest Asia. Sum of total ROH > 4 cM and number of total ROH > 4 cM are shown on the *x* and *y* axes, respectively. The baseline (red diagonal line) was computed using short ROH values (4 to 8 cM) in present-day West and Central Eurasian individuals to represent outbreed samples to determine the baseline. (C) is the zoomed version of (B) in which we draw the zoomed area with the gray rectangle. The red star denotes the only Çayönü individual, namely, cay007, that has more than 300,000 SNPs in the 1240K SNP Panel. The gray dots designate the ROH values for modern genomes.

Here, we asked whether PPN populations in the Fertile Crescent, which comprises the main domestication centers of animals and plants in Southwest Asia, also had low genetic diversity levels similar to the Central Anatolian PPN groups. Measuring genetic diversity using outgroup *f*_3_ values in genetic samples from Upper Mesopotamia (Çayönü), South Levant (Ain Ghazal), and Central Zagros (Ganj Dareh), we found that genetic samples from all three Fertile Crescent PPN settlements had higher diversity levels than those of the Central Anatolia PPN and are on a par with those of later-coming PN communities of Central and Western Anatolia (Fig. 5A and table S7). We note, however, that this relatively high within-population diversity does not seem to involve visible interindividual differences in ancestry proportions (e.g., variable Zagros ancestry) within the Çayönü sample but may be attributed to heterozygosity within the group (figs. S5 to S7 and note S4).

We next studied background population diversity through runs of homozygosity (ROH) analyses of one Çayönü genome (cay007) with sufficient coverage, using the hapROH algorithm (*36*), and compared these with ROH distributions estimated in other early Holocene Southwest Asian genomes (Materials and Methods). As reported earlier (*9*, *13*, *22*, *24*), Aşıklı, Boncuklu, and pre-Neolithic Caucasus genomes carried large numbers of ROH, indicative of a small population size. Certain Neolithic genomes (e.g., WC1 and Ash128) also showed a “right shift” when plotting the number versus sum of ROHs, indicative of recent inbreeding (Fig. 5, B and C) (*37*). In contrast, the cay007 genome had small and few ROHs, suggesting a lack of recent inbreeding and a relatively large population size, respectively, in line with outgroup *f*_3_–based analysis results.

Overall, these results suggest that the demographic transition observed in Central Anatolia between the PPN and the PN did not take place in the Fertile Crescent, at least not at the same magnitude. This observation is in line with radiocarbon-based estimates of low population density on the Central Anatolian plateau relative to other regions of Southwest Asia during the early Holocene (*38*). This result would also be consistent with the Taurus and Zagros Mountains supporting large hunter-gatherer populations, as well as the progenitors of plant and animal domesticates (*39*–*41*).

### Çayönü coburials reflect nuclear and possibly extended family structures

**Fig. 6.**
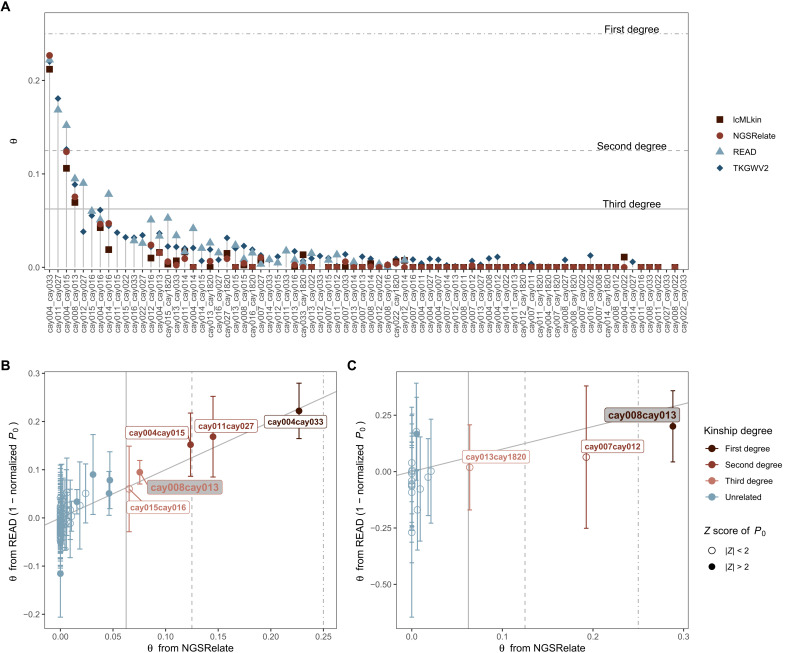
Kinship coefficient (θ) estimates among Çayönü individuals. Comparison of kinship coefficients inferred for 76 pairs using four different software [NGSRelate (*44*), READ (*45*), lcMLkin (*46*), and TKGWV2 (*47*)] is shown in (**A**). The figure only shows estimates when a pair had >2000 shared SNPs available in that analysis (which may differ for different software). (**B**) and (**C**) show autosomal and X-chromosomal estimates of θ, respectively, inferred from NGSRelate and READ. In the last two panels, NGSRelate θ estimates are shown on the *x* axes and READ θ estimates, calculated as (1 − normalized *P*_0_), on the *y* axes. Vertical bars represent ±2 SE of *P*_0_ values. Vertical dotted, dashed, and straight gray lines intersect with expected θ values for first-, second-, and third-degree relatives, respectively. Annotation with the gray label shows the pair cay008 and cay013. The full list of kinship coefficient estimates for all possible pairs is given in table S8.

**Fig. 7.**
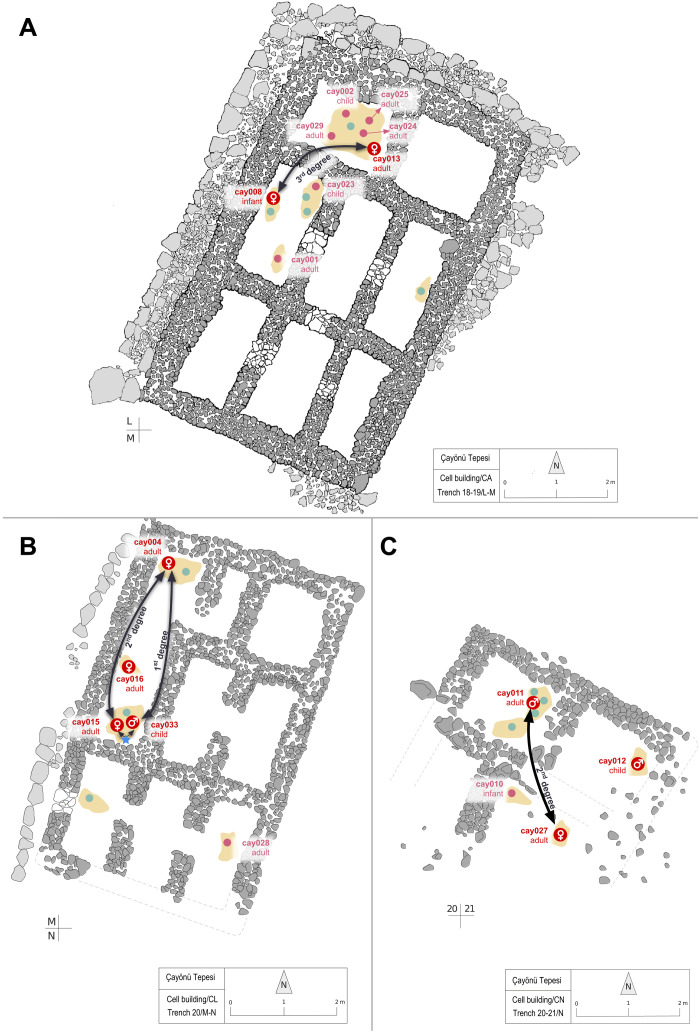
Locations of Çayönü coburials interred in domestic buildings. All three buildings belong to the cell building subphase. The figure shows plans of buildings coded (**A**) CA, (**B**) CL, and (**C**) CN. Red dots represent individuals analyzed in this study, pink dots represent individuals screened for aDNA but with insufficient preservation, and blue dots represent burials of other individuals within the same buildings. Black curved lines show the closely related pairs in each building and the estimated kinship level. The blue star in (B) indicates that the pair (cay015-cay033) is likely close genetic kin but were not reported as their number of shared SNPs was below the chosen threshold (<2000 SNPs) (Materials and Methods and table S8).

Recent work revealed that in the Central Anatolian PPN communities, Aşıklı Höyük and Boncuklu Höyük, coburials frequently included close genetic kin, suggesting that these earliest sedentary communities may have been organized around biological families (*13*), as hypothesized earlier (*42*, *43*). In contrast, coburials appear to have rarely comprised close genetic kin in the PN communities of Çatalhöyük and Barcın (*13*), implying a different pattern of kinship/social structure in the latter. However, the degree to which these observed patterns may be representative of their respective periods has remained uncertain because of the small sample sizes analyzed. Here, we investigated genetic kinship among Çayönü coburials using two approaches. First, using four different methods that have sensitivity of up to second- or third-degree relatedness (*44*–*47*), we estimated genetic kinship levels among a total of 76 pairs that had sufficient data, with 9 of these pairs representing coburials in the same buildings. We could identify four closely related pairs, including possible first-, second-, and third-degree relationships (Fig. 6, A to C, and tables S7 and S8) (Materials and Methods). This may be an underestimate, as possible close kin pairs with shared single-nucleotide polymorphism (SNP) numbers below threshold were not included (table S8). Notably, all these related pairs were interred in three buildings, and each pair shared the same building (Fig. 7).

We hypothesized that the nine individuals who were coburied with others but were not closely related could still belong to the same extended biological families. We investigated this by testing whether each of these coburied pairs was genetically closer to each other than to other Çayönü individuals, using outgroup *f*_3_-statistics. We found that coburied pairs who were not identified as close genetic kin were still slightly genetically closer to each other than pairs from distinct buildings (effect size = 0.03, permutation test, *P* < 0.001) (fig. S8). Nevertheless, we cannot fully distinguish between spatial and temporal effects given our small sample size (fig. S9); the question thus deserves being readdressed using larger samples with more intensive dating on skeletons.

Our results are largely similar to observations from the contemporaneous Aşıklı and Boncuklu of Central Anatolian PPN (*13*). Hence, biological family–based coburial cultures may belong to a diverse set of derived cultural traits shared among communities across Southwest Asia in this period. The social importance of Neolithic coburials and whether they represented household members remain ambiguous, although the observed patterns are consistent with the notion that biological family structures played a role in social organization in Southwest Asia during the Neolithic Transition (*42*, *43*). These results also render the reported deficiency of common genetic kinship among coburials in the PN communities of Barcın and Çatalhöyük ever more intriguing (*13*, *48*, *49*).

### A toddler of migrant descent, with artificial cranial deformation and cauterization

Our genetic comparisons highlighted a 1.5- to 2-year-old female toddler, cay008, as an outlier, with conspicuously higher genetic affinity to Zagros populations (Figs. 1D and 2 and fig. S3). Genetic kinship analysis using autosomal loci suggested a third-degree relationship between this individual and an adult female, cay013, interred in the same building (Figs. 6 and 7 and table S8). In contrast, analysis of their X-chromosomal loci indicated a genetic relationship closer than third degree (Fig. 6C and table S9). Such a discrepancy would be expected if cay013 was the paternal kin of cay008. Pedigree analysis suggested cay013 being the paternal great-aunt of cay008 as a possible scenario (Materials and Methods and figs. S10 and S11). In addition, the mitochondrial DNA haplogroup of cay008 (haplogroup T2g) was a clear outlier within the Çayönü sample, which consisted mostly of haplogroup K1 (table S3). These lines of evidence suggest that the Zagros-like ancestry of cay008 was inherited from her maternal side and her migrant ancestors bred with local individuals.

**Fig. 8.**
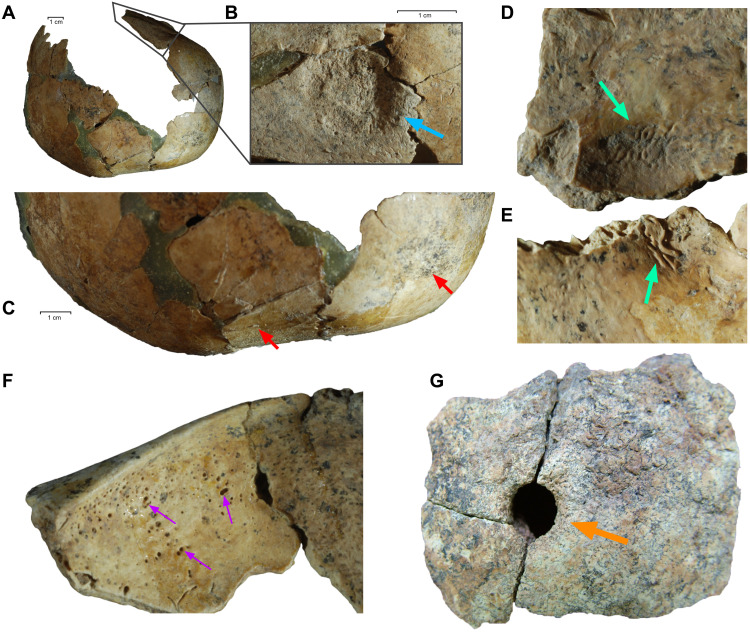
Cranial features of the cay008 toddler. (**A**) Frontal flattening, post-coronal depression, bulging on the parietal tuber, and fronto-occipital grooving suggest a double-bandaged circular-type cranial deformation. (**B**) Cauterization with a circular depression found on the post-coronal area on the left parietal bone. The bone is very thin in the center, and the edge of the lesion is elevated. (**C**) An enlarged picture of a post-coronal depression and frontal flattening. (**D** and **E**) Endocranial lithic lesions similar to serpens endocrania symmetrica on the occipital bone. (**F**) Slightly developed cribra orbitalia on the right orbital roof. This lesion together with porotic hyperostosis is mainly related to anemia. (**G**) Cranial trepanation performed by drilling on the skull of Çayönü individual ÇT’78 KE 6-2/3a SK5 (not represented in our genetic sample).

This toddler further displayed two intriguing features in her cranium (Fig. 8, A to F). First, cay008’s skull appears to be subject to artificial cranial deformation, or intentional head shaping, manifested as frontal flattening with a fronto-occipital groove and post-coronal depression (Fig. 8C). This could be produced by a double-bandaged circular head-shaping procedure. Three additional individuals in our sample also showed similar evidence, including cay013, the adult female relative of cay008 (table S1). Although circular head shaping with two bandages was previously documented in Neolithic Southwest Asia (*50*, *51*), Çayönü presents one of the earliest known examples of this tradition.

The cay008 skull also presents evidence of cauterization, i.e., the intentional burning of the cranium by an instrument (Fig. 8B). Cauterization marks were prevalent in Neolithic populations in Anatolia and Europe (*51*, *52*), but to our knowledge, cay008 shows the earliest documented case of this treatment. Cauterization marks from Europe are usually associated with trepanation, performed to thin the cranial bone (*53*), but the cranium of cay008 lacks a trepanation signal. Instead, we observed endocranial lesions reminiscent of serpens endocrania symmetrica on the inner surface of the fragmented occipital of cay008, suggesting that the toddler suffered from an infection (Fig. 8, D and E). The cranium also showed cribra orbitalia, which can signal anemia (Fig. 8F). We hypothesize that cauterization on the parietal bone might have been applied to treat the adverse effect of these diseases. The bone formation suggests that the toddler lived for a period of time after cauterization.

The evidence for cauterization, widespread head shaping, and additional reports of trepanation in Çayönü (Fig. 8G) altogether suggest a prominent culture of intentional body modification in this community (*54*). Body modifications may have developed in parallel with other aspects of cultural innovation in Çayönü and could also be shared interregionally; cases of artificial cranial deformation and trepanation are known from various Neolithic sites in the Fertile Crescent (*50*, *55*–*57*).

### The demographic impact of Upper Mesopotamia on Neolithic and post-Neolithic Anatolia

Last, we investigated the possible role of Upper Mesopotamia as a source of post-7000 BCE eastern gene flow into Anatolia. Eastern gene flow events have been inferred from increasing levels of early Holocene South Caucasus– and Zagros-related ancestry in Anatolian populations, starting by the PN and continuing into the Bronze Age (*9*, *14*, *58*, *59*). It was speculated that the original source of Caucasus/Zagros-related ancestry might be Upper Mesopotamia (*14*, *59*). This could be plausible given our results above, specifically that our Çayönü sample included >25% Zagros ancestry relative to Central Anatolian PPN populations (Fig. 2C and fig. S4). We thus asked whether the post-7000 BCE eastern admixture in Anatolian populations is better explained by gene flow from an early Holocene Caucasus–related group or from Upper Mesopotamia, represented by Çayönü. We computed *D*-statistics in the form of *D*(*Yoruba, CHG/Çayönü; X, Anatolia EP/Anatolia PPN*), where *X* was a Neolithic–to–Bronze Age population from Anatolia/Aegean, *CHG* represents early Holocene Caucasus (the so-called “Caucasus hunter-gatherers”), and *Anatolia EP/Anatolia PPN* represents Epipalaeolithic Pınarbaşı and PPN Boncuklu of Central Anatolia, respectively.

**Fig. 9.**
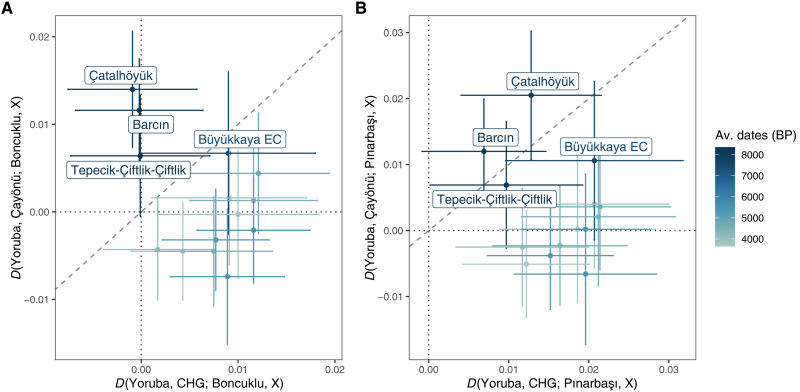
Biplots of *D*-statistics illustrating excess allele sharing between Çayönü and post-7000 BCE populations from Central/Western Anatolia. *D*-statistics were computed in the form of *D*(*Yoruba, pop1; pop2, X*), where *X* represents PN, Chalcolithic, and Bronze Age populations from the Anatolian Plateau. Each population is represented by a dot and error bars representing ±2 SE. The list of populations and *D*-statistics can be found in table S5. In both panels, *pop1* corresponds to CHG on the *x* axes, whereas on the *y* axes, *pop1* corresponds to the Çayönü population. *pop2* is represented by Boncuklu (Central Anatolian PPN) in (**A**) and Pınarbaşı (Central Anatolian EP) in (**B**) in both axes. The slope of the diagonal dashed line is 1 showing *x* = *y*, and the intercept of both vertical and horizontal dotted lines is 0.

This revealed two interesting results. First, we found that Çayönü genomes show higher genetic affinity to PN Anatolian populations Çatalhöyük, Tepecik-Çiftlik, and Barcın than to pre-7000 BCE Anatolian genomes (Fig. 9, A and B; fig. S12; and table S5). Moreover, this affinity was weak or absent when using CHG instead of Çayönü (*D*-statistics ≈ 0). This result is consistent with Upper Mesopotamia, but most likely not the Caucasus, being the source of eastern gene flow into Central Anatolia and possibly also into Western Anatolia around 7000 BCE. The finding also resonates with archaeological evidence from Çatalhöyük, where the mid-seventh millennium BCE witnesses the first introduction of obsidian from the Bingöl area of Eastern Turkey, the appearances of lithic types akin to “Çayönü tools,” and the growing use of the pressure technique in lithic industries (*60*–*62*).

Second, starting with early Chalcolithic in Anatolia, Çayönü genomes lose their affinity to post-Neolithic Anatolians, while the CHG sample gains affinity to post-Neolithic Anatolians over pre-7000 BCE Anatolians (Fig. 9, A and B). Hence, PPN Çayönü-related groups do not appear as the direct source of Caucasus-related ancestry in post-Neolithic Anatolia. This can be explained in two ways. One is that Caucasus-related influence in post-Neolithic populations emerged from a region other than Upper Mesopotamia, such as the South Caucasus, Zagros, or North Anatolia. An alternative scenario is that the Upper Mesopotamian gene pool itself changed after 7000 BCE by Zagros/Caucasus-related gene flow. In this case, Upper Mesopotamia could have remained the source of eastern gene flow into Anatolia with its new genetic profile.

Whereas the main driver behind European Neolithization has been recognized as mass population movements from Anatolia and/or Southeast Europe (*10*, *23*, *25*), the role of human movement in the multimillennia process of Neolithization in Southwest Asia is less understood. Here, we described the formation of Upper Mesopotamian PPN populations, represented by PPNB Çayönü, as an admixture event between western and eastern populations of early Holocene Southwest Asia. The PPNB Çayönü community appears to have carried relatively high genetic diversity levels relative to PPN Central Anatolia and pre-Neolithic Europe.

Nearly half a century ago, archaeologists Robert Braidwood and Halet Çambel described Çayönü as a perfect spot for the emergence of sedentism and agriculture, because of its location along the hilly flanks of the Taurus and Zagros Mountains where progenitors of plant and domesticates naturally coexisted (*63*). We hypothesize that Çayönü was also a lively hub of interregional networks, potentially because of its location between the sources of the Tigris and the Euphrates rivers in Upper Mesopotamia. Recent discoveries and ongoing research at sites such as Göbekli Tepe, Gusir Höyük, and Karahan Tepe (*2*, *64*, *65*) continue to demonstrate the importance of this region as a central node of cultural dynamism and social networks.

*Note added in proof:* Simultaneous with the acceptance of this manuscript for publication, an independent study that also included Upper Mesopotamian Neolithic genomes was published in *Science* (doi:10.1126/science.abq0762). The observations reported in the present manuscript regarding the mixed west-east ancestry of Upper Mesopotamian Neolithic communities are consistent with those reported in *Science*.

## MATERIALS AND METHODS

### Laboratory procedures

#### Sample collection and direct radiocarbon dating

Petrous bones from 33 human individuals from Çayönü, housed at the Hacettepe University in Ankara, Turkey, were used in aDNA experiments (Fig. 1A and Table 1). Table S1 provides archaeological and anthropological background information of all individuals. Eight of the deep sequenced samples were C14-dated with accelerator mass spectrometry at Türkiye Bilimsel ve Teknolojik Araştırma Kurumu Marmara Araştırma Merkezi [The Scientific and Technological Research Council of Turkey - Marmara Research Center (TÜBİTAK-MAM)] (Gebze, Turkey). Radiocarbon ages were calibrated using the IntCal13 calibration curve (*66*) with OxCal v4.2 (*67*).

**Table 1.** Description of sequenced Çayönü individuals. Asterisk denotes individuals used in population genetic analyses to represent the local Çayönü population.

**Excavation ID**	**C14 dates (cal BCE)**	**Sample ID**	**Average read length**	**Genome coverage (×)**	**Genetic sex**	**Note**
ÇT’78 S.16	7649–7538	cay004	51.44	0.08	XX	*
ÇT’81 S.2a	8496–8306	cay007	54.40	0.49	XY	*
ÇT’86 S.2	7842–7598	cay008	51.51	0.08	XX	Identified as genetic “outlier”
ÇT’78 S.7	7601–7524	cay011	52.56	0.04	Consistent with XY but not XX	*
ÇT’78 S.6	–	cay012	52.03	0.04	XY	*
ÇT’78 S.21	7882–7606	cay013	49.92	0.14	XX	*
ÇT’81 HB isolated	8211–7812	cay014	50.74	0.04	XX	*
ÇT’81 S.15	–	cay015	51.40	0.02	XX	Relative of cay004
ÇT’81 S.8	7882–7606	cay016	52.31	0.04	XX	*
ÇT’78 S.25	8300–8232	cay022	60.96	0.04	XX	*
ÇT’78 S.1	–	cay027	49.63	0.02	XX	Relative of cay011
ÇT’84 S.60	–	cay033	51.89	0.02	Consistent with XY but not XX	Relative of cay004
ÇT’70 S.13/ÇT’70 S.11b	–	cay1820	50.89	0.07	XX	*
						Identical individuals/ merged libraries

#### aDNA extraction, whole-genome library preparation, and sequencing

The experiments were carried out in dedicated aDNA facilities at the Middle East Technical University and Hacettepe University in Ankara, Turkey. To prevent contamination, all equipment and utensils were decontaminated with DNA AWAY or a bleach solution at each use and also in between handling the samples. Ultraviolet (UV)–insensitive solutions were UV-irradiated for 10 min at a distance of 5 cm before use. Negative controls were included at each step of the experiments to be able to track potential contamination originating from the reagents or handling the samples.

Before DNA extraction, outer surfaces of the petrous bones were scraped to a depth of c. 1 mm with single-use scalpels. The cochlea and the surrounding compact bone were cut out using the Dremel tool (*68*), and a piece from this region was ground into a fine powder using a SPEX 6770 freezer mill. About 120 mg of bone powder was transferred to a 2-ml screwtop tube. aDNA extraction was performed following the Dabney *et al.* (*69*) protocol. Two tubes of 80-mg hydroxyapatite, one at the beginning, the other at the end of each set of extractions, were used as negative controls. Blunt-end, double-stranded, Illumina-compatible sequencing libraries with double indexes were prepared using the Kircher *et al.* (*70*) protocol and sequenced on the Illumina NovaSeq 6000 platform using NovaSeq S1 flowcells at low coverage (median of c. 26 million reads per sample; table S3). After alignment (see the “Sequencing data preprocessing” section below), 14 individuals’ libraries were found to contain >0.3% endogenous DNA; these were further sequenced on the Illumina NovaSeq 6000 platform using S1 flowcells.

### Quantification and statistical analysis of raw data

#### Sequencing data preprocessing

We removed Illumina adapter sequences in fastq files and merged the paired-end sequencing reads using AdapterRemoval v2.3.1 (*71*), requiring an overlap of at least 11 bp between pairs. The merged reads were mapped against the Human Reference Genome (hs37d5), using the “samse” command of BWA aln v0.7.15 (*72*) with the parameters “-n 0.01 -o 2” and with seeding disabled using the “-l 16500” option. Polymerase chain reaction duplicates were removed using the FilterUniqueSAMCons.py script by collapsing the reads with identical start and end positions (*73*). Last, we filtered reads shorter than 35 bp in length and with more than 10% mismatches to the reference genome. Multiple libraries belonging to the same individual were merged using SAMtools merge v1.9 (*72*), and duplicates were removed again with the same filtering procedure. We also remapped published ancient genomes following the same procedures for comparative analysis (table S4). Reads obtained from libraries were trimmed from both ends by 10 bp using the trimBAM command of bamUtil software (*74*) to remove postmortem deamination artifacts.

#### Contamination estimates and genetic sex determination

Postmortem deamination patterns were estimated from pretrimmed data using PMDtools (*75*) with the “--deamination” parameter. We conducted authenticity analysis using three additional approaches: contamMix (*76*) and Schmutzi (*77*), which make use of the rate of consensus mitochondrial sequence mismatches, and ANGSD, which estimates the excess of heterozygous positions for the haploid X chromosome in male individuals (*78*). To detect nonendogenous reads, the contamMix library in R calculates a contamination probability using a reference panel of 311 diverse mitochondrial genomes. For this approach, consensus mitochondrial sequences were created using ANGSD (*78*) with parameters “-doFasta 2 -doCounts 1 -minQ 30 -minMapQ 30 -setMinDepth 3 -r MT.” Schmutzi also calculates probability of authenticity using deamination patterns on the consensus mitochondrial DNA fragments, but it additionally includes the information from read lengths and postmortem deamination since the longer and nondeaminated fragments are potential contamination sources (*77*). Last, for the male individuals belonging to our sample set, contamination based on X chromosome was estimated by running the ANGSD (*78*) algorithm with the command “angsd -i BAMFILE -r X:5000000 -154900000 -doCounts 1 -iCounts 1 -minMapQ 30 -minQ 30” for X chromosome–mapped reads. The probability of heterozygosity on the X chromosome was then calculated using the R script contamination.R (*76*) and the reference files provided in the ANGSD package (*78*). To determine the genetic sex of ancient individuals, we used the script ry_compute.py (*79*), which computes *R*_Y_, the ratio of the number of reads mapped to the Y chromosome to the number of reads mapped to both the X and Y chromosomes per BAM file.

#### Uniparental markers

Y-chromosome haplogroups of male individuals (*n* = 4) were assigned using the “best path” method of pathPhynder (*80*), which adds particularly low-coverage samples on phylogenetic trees annotated with haplogroups. We used the compiled Y-chromosome dataset and phylogenetic tree in (*80*). By counting the number of derived and ancestral alleles represented on each branch of the tree, we determined the best path for each male individual. Then, we assigned the relevant haplogroup according to the node of the sample on the tree (table S3). We used default parameters of pathPhynder. We visualized the tree using iTOL v6 (fig. S13) (*81*). Because of missing data among informative SNPs, we could determine only the basal branch “CT” for two individuals (cay012 and cay033). While cay011 was placed onto the haplogroup G branch (supported by 10 derived variants above the branch and 2 derived variants at the assigned branch), the cay007 individual was assigned J2a1a (supported by 152 derived variants above the branch and 4 derived variants at the assigned branch).

Consensus mitochondrial sequences were produced from the sequence alignment files using ANGSD (*78*) with parameters “-doFasta 2 -doCounts 1 -minQ 30 -minMapQ 30 -setMinDepth 3.” Breadth of coverage was calculated for the positions with depth ≥ 3 (table S3). Then, we identified haplogroups for individuals for which we recovered more than 15% of the mitogenome, using HaploGrep (*82*).

#### SNP dataset preparation

After remapping previously published ancient genomes with the above-described procedure (see the “Sequencing data preprocessing” section above), SNP calling was performed in two steps: (i) Pileup files were generated with the SAMtools (v.1.9) mpileup software (*83*), and (ii) pseudo-haploid genotypes were generated by randomly choosing one allele per SNP locus from all BAM files using the PileupCaller v1.4.0.5 tool (https://github.com/stschiff/sequenceTools) and the option “-randomHaploid.” In this study, we used three different SNP panels. Because the Human Origins Dataset has a relatively limited number of SNPs (~600 K) but covers wider modern diversity than other datasets, we chose it for analyses of the type PCA (principal components analysis) and ADMIXTURE. On the other hand, we used the 1240K Dataset in hapROH analysis, which has been optimized using this dataset. Last, since the 1KG Yoruba Dataset includes more SNPs in total, we chose this for analyses such as *D* tests and kinship estimation, where higher SNP numbers could partly alleviate the low depth of coverage of our sample.

##### Human Origins Dataset

Present-day Western Eurasian samples were extracted from the Human Origins Dataset (*11*, *26*) and merged with published Southwest Asian Early Holocene genomes (table S4) and the newly generated Çayönü samples. A total of 605,775 SNPs were included. This dataset was used to perform the PCA and ADMIXTURE analysis.

##### 1240K Dataset

We downloaded the Allen aDNA Resource (https://reich.hms.harvard.edu/allen-ancient-dna-resource-aadr-downloadable-genotypes-present-day-and-ancient-dna-data) (v50.0) 1240K Dataset and extracted Human Genome Diversity Project high-coverage genomes. After calling these positions in Southwest Asian Early Holocene genomes and Çayönü samples, we merged the datasets. The dataset consisted of 1,151,145 autosomal positions to compute ROHs.

##### 1KG Yoruba Dataset

We generated another SNP panel choosing biallelic sites with minor allele frequency (MAF) greater than 10% in the African Yoruba genome sample (56 females and 52 males, a total of 108 individuals) from the 1KG phase 3 dataset (1000 Genomes Project Consortium, 2015). We merged this with genotype files generated from high-coverage genomes (*n* = 279) from the Simons Genome Diversity Project (SGDP) representing present-day global diversity (*84*). In addition, we included published ancient genomes from Western Eurasia (table S4) and the Çayönü population. After merging the datasets, the remaining 5,991,735 autosomal SNPs in total were included. We conducted all genetic kinship and population genetic analyses (other than PCA, ADMIXTURE, and ROH) on this dataset. We also prepared another dataset including 220,384 X-chromosomal SNPs with the same procedure by removing the pseudoautosomal regions to conduct kinship analysis based on the X chromosome.

#### Genetic kinship analyses

To determine close kin up to third-degree relatedness, we estimated genetic kinship coefficients (θ) between each pair of individuals. To achieve this, we ran four alternative software [READ (*45*), NGSRelate v2 (*44*), lcmlkin (*46*), and TKWGV2 (*47*)] and jointly analyzed their results. READ calculates pairwise mismatch rates (*P*_0_) of pseudo-haploid genome pairs in 1–million base pair (Mbp) windows and normalizes these values with the median *P*_0_ of the sample, assuming that the median represents a nonrelated pair (*45*). We used (1 − normalized *P*_0_) values as an estimate of the kinship coefficient (θ). NGSRelate v2 (*44*) calculates nine Jaccard coefficients from genotype likelihoods, using background allele frequencies, and then computes θ = *J*_1_ + 0.5 × (*J*_3_ + *J*_5_ + *J*_7_) + 0.25 × *J*_8_. Since our Çayönü genome sample was composed of low-coverage genomes, to increase the resolution of NGSRelate, lcmlkin, and TKGWV2, we provided background allele frequencies calculated from a total of 211 Southwest Asia Holocene individuals including Çayönü and published Anatolia, Levant, and Zagros genomes. All four methods yielded consistent results in many pairs, although READ appears to overestimate θ values relative to other methods in low-coverage pairs.

Of the 14 genomes produced, one pair (cay018 and cay020) were coherently identified as “identical/twin” by all methods. These two libraries were obtained from human remains from the same building, and one library was constructed from a left-side and the other one from a right-side petrous bone. The reconstructed skull showed that both petrouses could belong to the same infant. In addition, both libraries showed similar DNA preservation patterns (see table S3 for human proportions and damage patterns), which would be consistent with the possibility that the bones derived from a single individual. We thus merged these two libraries, leaving us with a total of 13 individuals.

After merging cay018 and cay020, we repeated the genetic kinship analysis with the 13 individuals but using autosomal and X-chromosomal data separately. To increase confidence in the estimates, we stipulated a minimum number of overlapping SNP counts between pairs of individuals, >2000 SNPs when analyzing autosomal data and >200 SNPs when analyzing X-chromosomal data. We further required a significance of |*Z*| > 2 for the normalized *P*_0_ in READ analysis (estimated using variance among the genome-wide 1-Mbp windows by the READ software). All methods yielded well-correlated kinship coefficients (in the comparison of distinct approaches READ and NGSRelate; Spearman’s rho for autosomal θ = 0.65 and for X-chromosomal θ = 0.49). One pair (cay004-cay033) was estimated as first-degree kin, two pairs (cay004-cay015 and cay011-cay027) as second-degree kin, and one pair (cay008-cay013) as third-degree kin according to autosomal kinship analysis. All these related pairs were coburied in the same buildings. We excluded one of each pair of close kin (the lower-coverage individual of each pair) from downstream population genetic analyses to ensure sample independence. Notably, cay008 and cay013 individuals were closer kin than third degree according to X-chromosomal kinship analysis. Both individuals were females; while cay013 was an adult, cay008 individual was a 1.5- to 2-year-old child (table S1).

To resolve the pedigree of the relationship between cay008 and cay013, we constructed and analyzed possible maternal and paternal pedigrees of cay008 using the pedsuite package in R (*85*). Since she died before fertile age, cay008 cannot be the ancestor but could only be related to cay013 through her parents. Given this, we generated all possible third-degree relatives (great-grandparents, great-aunts, half-aunts, and first cousins). Then, we calculated theoretical values of autosomal and X-chromosomal θ values for all pairs in the pedigrees (figs. S10 and S11).

We note that 2 of 78 pairs had <2000 autosomal overlapping SNPs and thus have not been reported in the main text (table S8). One of these, cay015-cay033, were coburied in the same building, showing a signal of being third-degree related, which was supported by their shared close kinship to cay004. The other pair, cay015-cay027, were buried in different buildings (Fig. 6).

#### *f*- and *D*-statistics

We computed outgroup *f*_3_-statistics and *D*-statistics using the qp3pop and qpDstat programs, respectively, from the ADMIXTOOLS package (v7.0.2) (*26*) with default parameters. We used the present-day West African Yoruba sample (*n* = 3) from the SGDP dataset (*84*) as an outgroup in both analyses. We ran pairwise outgroup *f*_3_-statistics for each individual from early Holocene Anatolia, Levant, Zagros, and Upper Mesopotamia. We corrected *P* values for multiple testing using the R “p.adjust” function’s false discovery rate (i.e., Benjamini-Hochberg) correction (*86*). In *D*-statistics involving Çayönü as a population, we performed analyses separately using the nine genomes excluding one individual from close relatives and excluding cay008.

#### Isolation-by-distance analysis and within-population diversity

We computed geodesic geographic distance using the geodist package in R (*87*). To test isolation by distance, we computed the model fit with the “lm” function in R and compared geographic distance and genetic distance. We used 1 − *f*_3_ scores of each individual pair as a proxy for genetic distance. We filtered out pairs from the same site, pairs with a >1000-year time difference between individuals, and pairs with <2000 overlapping SNPs.

To compare within-population genetic diversity of specific Neolithic settlements from Southwest Asia, we again used 1 − *f*_3_ scores between pairs of individuals as a measure of genetic difference. We calculated significance using permutation tests through an in-house R script, where we randomized population identity with the R “sample” function.

#### ROH analysis

We used the Python package hapROH v.0.3a4 (https://pypi.org/project/hapROH/0.3a4/) to detect ROHs, which are long, homozygous stretches of the genome that result from the common ancestry of the maternal and paternal chromosomes (*37*). We used the default parameters of hapROH with pseudo-haploid genotypes, which contain more than 300,000 SNPs of the 1240K SNP Panel, the default genetic map of hapROH, and 5008 global haplotypes from the 1000 Genomes Project (*36*, *88*). We detected ROH for ancient genomes from Early Holocene Southwest Asia (table S4) and West and Central Eurasian present-day genomes (*89*). We performed linear regression using short ROH [4 to 8 centimorgan (cM)] in present-day genomes to create a baseline that represents solely drift with no recent inbreeding. Right shift from the baseline indicates that parents of this individual could be close kin, whereas individuals that are around the baseline and have a relatively high number of ROH come from a population with potentially low *N*_e_ (effective population size) (*37*). We filtered out ROHs < 4 cM following the original hapROH publication, which suggested that the method can detect ROH > 4 cM (*36*).

### Dimensionality reduction analyses

#### Multidimensional scaling

We summarized the outgroup *f*_3_-statistics calculated across all pairs of individuals using MDS and visualized the first two dimensions. First, we created a dissimilarity matrix of pairwise genetic difference (1 − *f*_3_) values. From this, we filtered out the pairs that had <2000 overlapping SNPs. We then applied the “cmdscale” function in R.

#### Principal components analysis

To perform PCA, we used the “smartpca” (version 16000) software of EIGENSOFT (v7.2.1) (*90*) with the “shrinkmode: YES” and “lsqproject: YES” option to project ancient individuals onto principal components calculated on genome-wide polymorphism data of 55 Western Eurasian present-day populations (760 individuals) from the Human Origins SNP Panel (*11*, *30*). We additionally computed elliptical confidence for Çayönü individuals with the “ellconf: 0.95” function implemented in smartpca.

#### ADMIXTURE analysis

We performed unsupervised model-based cluster analysis using ADMIXTURE version 1.3.0 (*91*). We estimated ancestry components of present-day populations in the Human Origins SNP Array Dataset after pruning for linkage disequilibrium and filtering sites with MAF less than 5% in PLINK (www.cog-genomics.org/plink/1.9/) with parameters “--indep-pairwise 200 25 0.4” and “—maf 0.05,” which retained 179,175 SNPs. After filtering, we selected Western Eurasian modern-day populations (*n* = 629) and merged them with ancient individuals (*n* = 307), similar to (*92*). We performed clustering from *K* = 2 to *K* = 6 with default fivefold cross-validation (“--cv = 5”) and 10 replicate runs with different random seeds. The cross-validation procedure of ADMIXTURE was used to choose the optimal value for *K*. The LargeKGreedy algorithm of CLUMPP (*93*) was used to determine the common signals between each independent run.

#### Admixture modeling

We modeled admixture proportions using the “qpAdm” software from the ADMIXTOOLS (v.7.0.2) package. We selected a reference differentially related to left populations covering modern and ancient diversity (*94*). We found that the following base reference set was able to distinguish our relevant populations: *Mbuti*, *Ust_Ishim*, *Kostenki14.SG*, *MA1*, *Han*, *Papuan*, *Dai*, *Chukchi*, *Mixe*, *CHG*, *Natufian*, *WHG*, *AfontovaGora3*, and *Iberomaurusian*.

We then performed all possible two- and three-way models, adding published genomes representing late Pleistocene and early Holocene populations of Central Anatolia, Zagros, and Levant as surrogates (“left populations”) and Çayönü genomes as targets. We ran all qpAdm analyses with “allsnps: YES” option, which is robust to low-coverage data (*94*). Any model without a Central Anatolia–related source did not work, yielding *P* values <0.05. To test potential sex-biased admixture in the Çayönü population, we repeated the same analyses with the X-chromosome dataset. However, all runs failed most likely because of the low coverage of our Çayönü data, combined with the relatively small number of X-chromosome SNPs (220,384 SNPs).

In addition, we modeled Anatolia PN populations, Barcın and Çatalhöyük, as a mixture of Anatolia PPN, Çayönü, and S Levant N population since there is a signal of admixture from Çayönü into these populations (table S6). To increase the resolution, we added an Anatolian Epipaleolithic individual into the reference set. Here, we only used shotgun sequenced published genomes from Barcın to avoid technical confounding.

#### Date comparisons

We compared summed probability distributions of C14-dated individuals using the “stackspd” function from the “rcarbon” package in R (*95*), with a window size of 100 years (fig. S9A). To test temporal overlap among individuals buried in cell building structures, we sampled ages from the calibrated probability distributions 10,000 times for each individual using the “sampleAges” function from the Bchron package in R (*96*) and computed differences of dates for each pair. Then, we calculated the mean difference and 95% quantiles to test whether individuals may have lived in the same time period or not (fig. S9B).

#### Visualization

All plots were generated in R (*97*) using ggplot (*98*) and ggpubr (*99*) packages. Other packages used to analyze, clean, and visualize the data are the following: tidyverse (*100*), patchwork (*101*), reshape2 (*102*), ggplotify (*103*), ggrepel (*104*), emojifont (*105*), ggforce (*106*), rgdal (*107*), raster (*108*), plyr (*109*), MetBrewer (*110*), and pedsuite (*111*).

## Supplementary Materials

### This PDF file includes:

Notes S1 to S5 Figs. S1 to S13 Table S1 References

## Notes

View/request a protocol for this paper from *Bio-protocol*.
