# Supplementary material for: A genomic snapshot of demographic and cultural dynamism in Upper Mesopotamia during the Neolithic Transition
Source: Sci Adv. 2022 Nov 4;8(44):eabo3609. doi: 10.1126/sciadv.abo3609 (PMC9635823; doi:10.1126/sciadv.abo3609)
Supplement: Supplementary file 1 — Notes S1 to S5 Figs. S1 to S13 Table S1 References [file sciadv.abo3609_sm.pdf]

Science Advances
AAAS

Supplementary Materials for

A genomic snapshot of demographic and cultural dynamism in Upper Mesopotamia during the Neolithic Transition

N. Ezgi Altınışık *et al.*

Corresponding author: N. Ezgi Altınışık, ezgialtinisik@hacettepe.edu.tr;
Yılmaz Selim Erdal, yserdal@hacettepe.edu.tr; Aslı Erim Özdoğan, aslierim09@gmail.com;
Füsun Özer, fusunozer@hacettepe.edu.tr; Mehmet Somel, msomel@metu.edu.tr

Sci. Adv. **8**, eabo3609 (2022)
DOI: 10.1126/sciadv.abo3609

The PDF file includes:

Notes S1 to S5
Figs. S1 to S13
Table S1
Legends for tables S2 to S9
References

Other Supplementary Materials for this manuscript includes the following:

Tables S2 to S9

Supplementary Note 1

A number of archaeogenomic studies have suggested that early Neolithic populations in the Fertile Crescent (or the Core Region of Neolithization) were largely descendants of local pre-Neolithic groups in the same regions, and likewise, that Ceramic / Pottery Neolithic (PN) groups were largely descendants of local Aceramic / Pre-Pottery (PPN) groups. First, the study by ref. (*12*) compared genomes from Epipaleolithic/Mesolithic and PPN groups in the Levant and in Zagros. In both regions the authors showed limited change in ancestry components between the pre-Neolithic and PPN. Second, ref. (*9*) and ref. (*10*) also suggested large-scale continuity between Aceramic and Ceramic populations in Central Anatolia, in line with archaeological evidence (*113*). Third, ref. (*7*) showed that 90% of Boncuklu (Central Anatolia PPN) ancestry can be traced back to Pınarbaşı (Central Anatolia Epipaleolithic). Fourth, work by ref. (*13*) estimated 89-92% of the Ceramic Central Anatolia (Çatalhöyük) gene pool derived from local Aceramic populations (Aşıklı and Boncuklu). Overall, the evidence points towards limited interregional mobility during Neolithization in Southwest Asia, in contrast to the Neolithization process of Europe which appears mainly driven by migrating Neolithic groups.

Supplementary Note 2

Several common features characterise the PPN sites of Upper Mesopotamia. Affinities of the PPNA community in Çayönü to contemporary sites in the Upper Tigris and Northwest Zagros regions have previously been noted (*16*). A large number of sites in these regions seem to have hosted sedentary hunter-gatherers living in lightly built, round or oval structures with semi-subterranean floors, including Çayönü, Demirköy, Hallan Çemi, Körtik Tepe, Nemrik, M'lefaat, and Qermez Dere (*114*, *115*). Further to the west, groups in the Euphrates valley and surrounding areas constructed round buildings in the PPNA as well, but have placed a more apparent emphasis on monumentality (e.g., Göbeklitepe, Jerf el-Ahmar, Tell 'Abr 3; (*116–118*)) that is lacking in the earlier phases of Çayönü. Yet noteworthy among the PPNA chipped stone assemblage of Çayönü is the presence of Nemrik and El-Khiam points (*16*, *119*, *120*), which are also known from Middle Euphrates, Upper Tigris, and Southern Levant, in sometimes overlapping distributions (*121*). Nemrik points, especially, appear to be a preferred arrowhead form from the Upper/Middle Euphrates to the Northwest Zagros, coinciding with the distribution of the so-called stone sceptres or batons (*116*, *122*, *123*). Subsistence-wise, the same region exhibits diversity with respect to the range of plant resources targeted, and intensive exploitation of crop progenitors is observed in some sites, but not universally across the region (*2*). Moreover, the main (wild) animal species consumed appears variable from site to site (e.g., pigs in Çayönü, sheep in Hallan Çemi, gazelle in Göbeklitepe, etc.), most likely depending on what was most readily available in the local environment (*18*). Intramural burial practices are another notable feature of PPN Upper Mesopotamia as well as Neolithic Southwest Asia. In the PPNA intramural subfloor burials are common, while in the following PPNB period more complex mortuary treatments (skull removal and sometimes plastering, bone caches and secondary burials) gain popularity, and buildings dedicated to multiple burials are known from sites including Çayönü in Upper Tigris, Dja'de el-Mughara and Abu Hureyra in Middle Euphrates, and Shkārat Msaied and Kfar Ha'Horesh in Southern Levant (*124*).

The PPNB also witnesses the shift from circular to fully rectangular buildings over a large portion of the Near East. Çayönü is one of the sites where this architectural transition can be tracked (*125*), along with Jerf el-Ahmar in Middle Euphrates (*126*), Tell Qaramel in Northern Levant (*127*), Jericho in Southern Levant (*128*), and (albeit at a later date than the others) Aşıklı in Central Anatolia (*129*). Communal buildings are known from the Levant, Upper Mesopotamia, and Central

Anatolia, but are mostly absent from Central Zagros. Two common threads between the PPNB rectangular special purpose structures in Upper Mesopotamia is the plastered floors and use of stone pillars in comparable arrangements, attested in sites such as Çayönü, Boncuklu Tarla, Göbeklitepe, and Nevalı Çori (*130*, *131*). Byblos points and "Çayönü tools" likewise make an appearance in Çayönü in this period (*16*). Former type of projectiles is among the hallmark artefacts of the Levantine PPNB (*132*), while Çayönü tools are distributed mainly in the Khabur and Tigris basins to the east (*121*). Another development in the PPNB is the introduction of pressure and bidirectional blade detachment techniques in Çayönü. The pressure technique appears to have spread to the Near East through either Central Asia or the Caucasus and in any case was present in PPNA Northwest Zagros, and is first seen in Çayönü in the early PPNB (*133*, *134*). Bidirectional blade technology, on the other hand, originated in PPNA Middle Euphrates, and spread in the Levant to become one of the key components of the entire region's PPNB assemblages (*135*). Introduced to Çayönü as imported products at first, it was only in the late PPNB that this technique started to be applied locally (*136*). Cereal and legume crop progenitors also appear across Southeast Anatolia in the early PPNB, some of them (Çayönü and Nevalı Çori) with early signs of domestication (*2*). The main faunal trend in the same region is the dramatic acceleration of sheep and goat exploitation, joining cattle and pigs in a trajectory that led to their domestication in the PPNB (*18*). Upper/Middle Euphrates and Upper Tigris PPNB communities with these four domestic livestock species contrast with both Southern Levant, Central Anatolia, and Central Zagros, where the same "barnyard complex" was completed only at later periods (*5*).

Çayönü retains a long and uninterrupted sequence from the beginning of the PPN to the emergence of pottery during the latter part of the Neolithic. Even though the archaeological remains exhibit significant dynamism and external interaction within this lengthy occupation, there is no archaeological evidence for complete population turnover(s) that might have resulted in these changes. Instead, the archaeological record shows a continuous development through time, likely local responses to both local and non-local contingencies. In this regard, it is possible to surmise that the genetic composition of the PPNA occupants of Çayönü was not radically different from the main profile identified among the sampled PPNB individuals from the site. In light of the similarities in material culture and the spatial proximity, such a genetic profile might also apply to the broader Upper Tigris region in earlier time periods. Yet it becomes harder to evoke such arguments as one moves further east following the Tigris and its tributaries and further west towards the Euphrates. Even though parallel archaeological patterns can be detected in Upper/Middle Euphrates and Northwest Zagros, communities in these regions were also susceptible to further genetic interchanges with the Levant, Anatolia, and Central Zagros, most readily visible in the networks of obsidian transmission that they were entangled in (*30*, *137*).

Supplementary Note 3

Among the four Fertile Crescent populations studied (Levant, C Anatolia, U Mesopotamia, Zagros), the differentiation between Zagros and the other groups was found to be higher than what would be expected simply from isolation-by-distance, which we interpret as "lowest effective migration" between Zagros and Upper Mesopotamia. This east-west differentiation is estimated using the outgroup-f_3 statistic calculated between pairs of individuals from different groups in the form of f_3(outgroup; popA, popB). This is theoretically not affected by strong drift (bottlenecks) within groups, but reflects average split time between groups (*26*). Our f_3 analysis thus suggests earlier split times between Zagros populations and other "Fertile Crescent" groups, relative to split times among the latter populations (i.e. Anatolia, Levant, as well as Çayönü). Moreover, the difference cannot be fully explained by geographic distance between Zagros and the other populations. We

refer to this pattern as east-west differentiation. Importantly, the observed differentiation pattern does not require population isolation during the Neolithic. In fact, the Çayönü genomes indicate the contrary, such that in Çayönü we find both east and west Fertile Crescent ancestry. Instead, the east-west differentiation is likely the remnant of earlier population isolation, *e.g.,* isolation during the LGM. Although post-LGM population admixture may have partly erased this differentiation signature, it can still be observed in our early Holocene genomic data.

Supplementary Note 4

The genetic diversity differences between two populations, say higher diversity in population A than population B, can arise through different means: (1) population A may carry higher inter-individual variability in its ancestry proportions than B (e.g. as ref. (*138*) has described for ancient Rome); (2) within-population ancestry proportions may be equally homogenous between A and B, and (2a) population A may be composed of admixture between genetically more distinct groups, and thus each individual will carry higher heterozygosity, or (2b) population A may have higher effective population size, and thus higher heterozygosity than B. The *qpAdm* analyses suggest that among these possible mechanisms, not (1) but (2a) and (2b) may contribute to higher diversity estimates in Çayönü compared to Boncuklu or Aşıklı (Fig. 5A).

Supplementary Note 5

We use "Anatolia" here following the traditional geographic definition, referring to the west of the Anatolian Diagonal, which is defined as the line running from southwest to northeast Turkey.

Fig. S1.
Human proportion and genomic coverage of the deep-sequenced genomes from Çayönü.

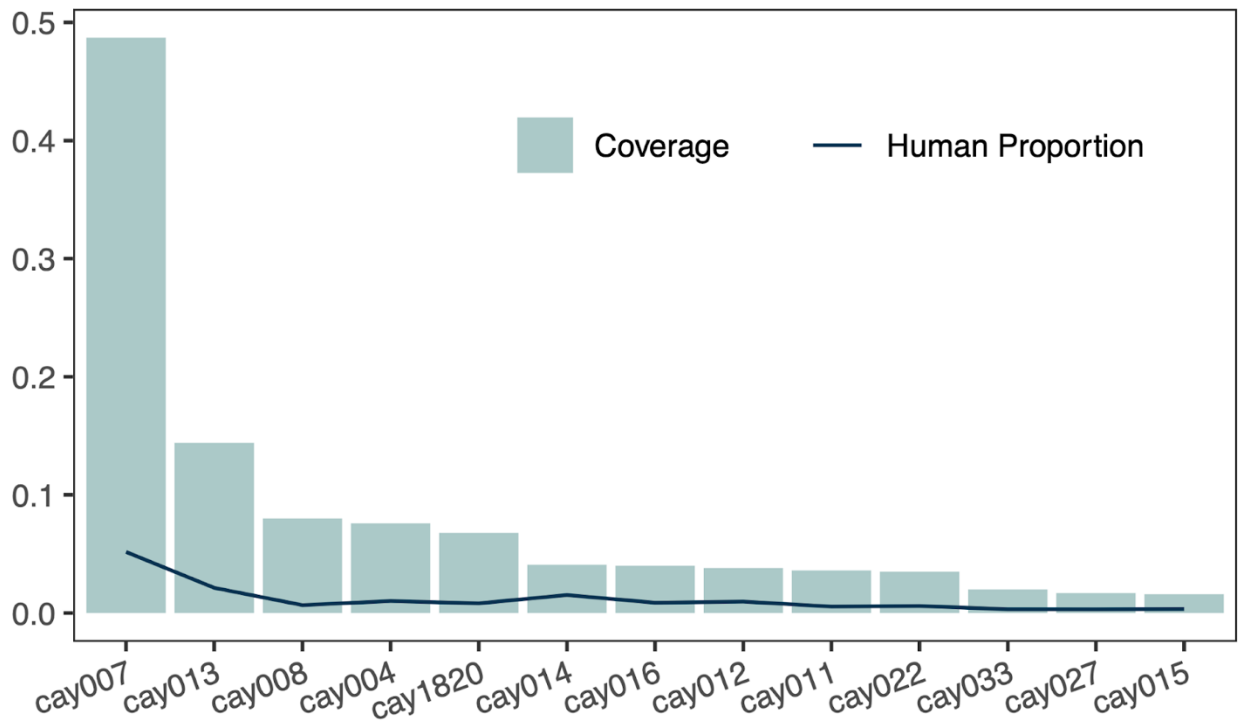

Fig. S2.

Comparison of the DNA preservation in Çayönü and its contemporaneous Central Anatolian sites, Aşıklı and Boncuklu.

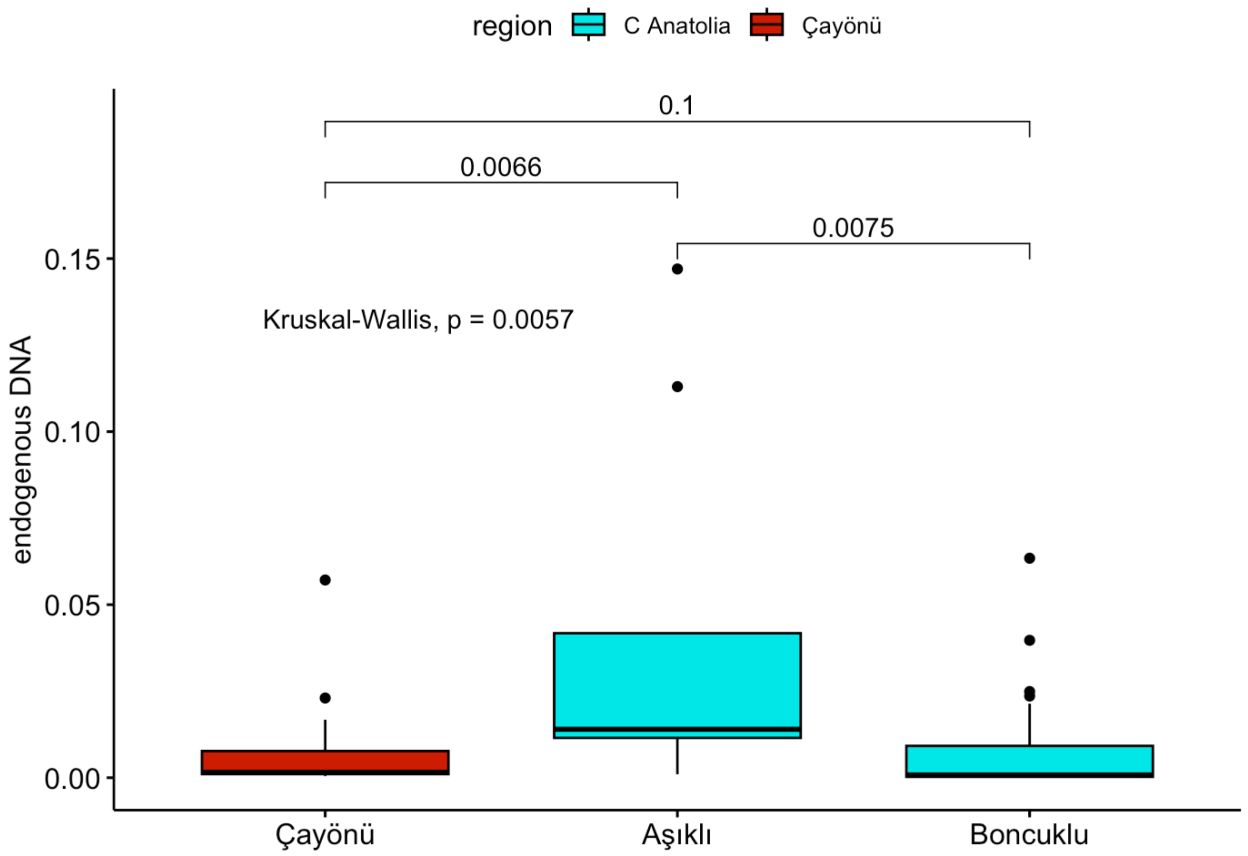

Fig. S3.

Principal Component Analysis (PCA) of Southwest Asian early Holocene populations. The ancient genomes (coloured squares and circles, with Çayönü genomes shown with red stars) were projected onto principal components calculated using 55 present-day West Eurasian populations (grey dots) (Table S4). We computed 95% confidence intervals using the ellipse function implemented in *smartpca* (*90*) for all Çayönü individuals and highlighted ellipses of two individuals, cay008 and cay015, also labelled in the figure. We note three points: (a) a Southern Levant Neolithic individual (KFH2_KFH002) falls into the Anatolian cluster, which is consistent with earlier observations (*7*). (b) The cay015 genome appears in the Southern Levant cluster. However, it has the lowest coverage in our sample (only 6,986 SNPs available to this analysis) and its 95% confidence range overlaps with most of Çayönü individuals. (c) The cay008 genome stands out as an outlier, such that its range overlaps with nearly none of the individuals belonging to the main Çayönü cluster.

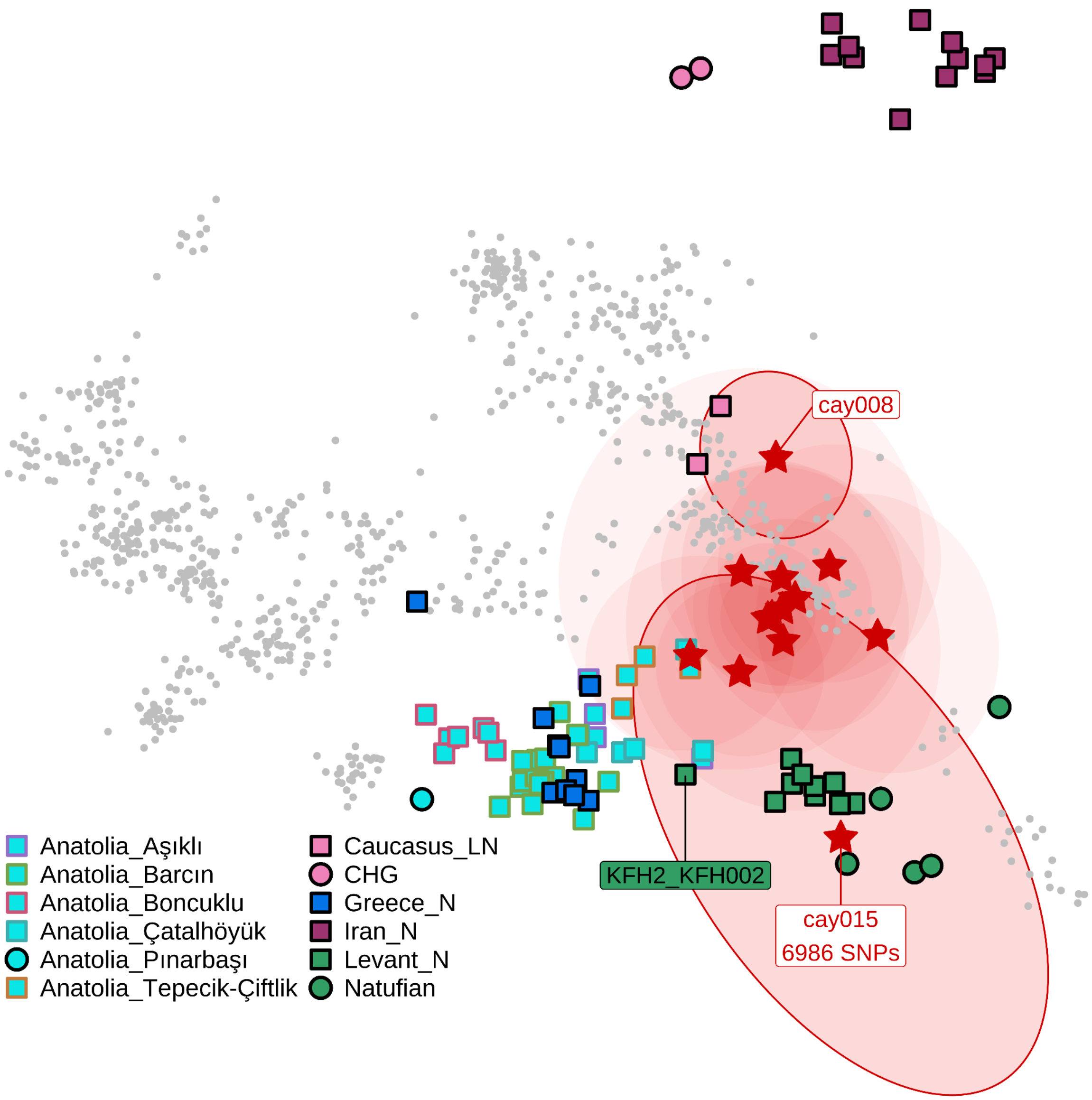

Fig. S4.

Modelling Çayönü with Zagros or Caucasus sources. The figure shows the results of feasible qpAdm models of the Çayönü core group (9 individuals) and cay008 genomes. Each line represents a model. These include either Zagros_N (Central Zagros Neolithic) or CHG (South Caucasus pre-Neolithic) genomes as source, in addition to Anatolia EP (Epipaleolithic) or Anatolia PPN, and South Levant Neolithic. All models are feasible with p>0.01. The full results are presented in Table S6.

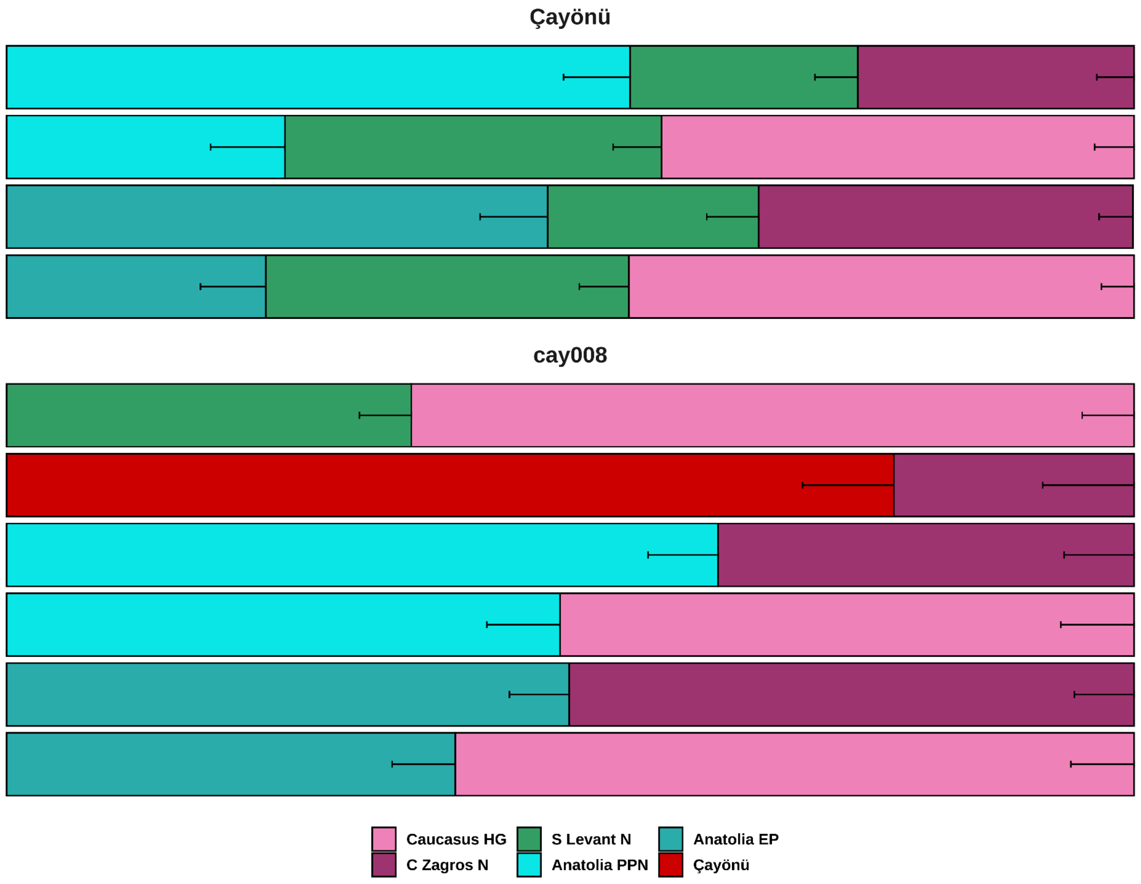

Fig. S5.
ADMIXTURE analysis of modern-day West Eurasian **(A)** and ancient Southwest Asian **(B)** genomes. We note that we excluded one of the individuals from each close kin pair in ADMIXTURE, to avoid creating a bias in allele frequencies (e.g. (*91*)). K=3 yields the lowest cross validation (CV) error.

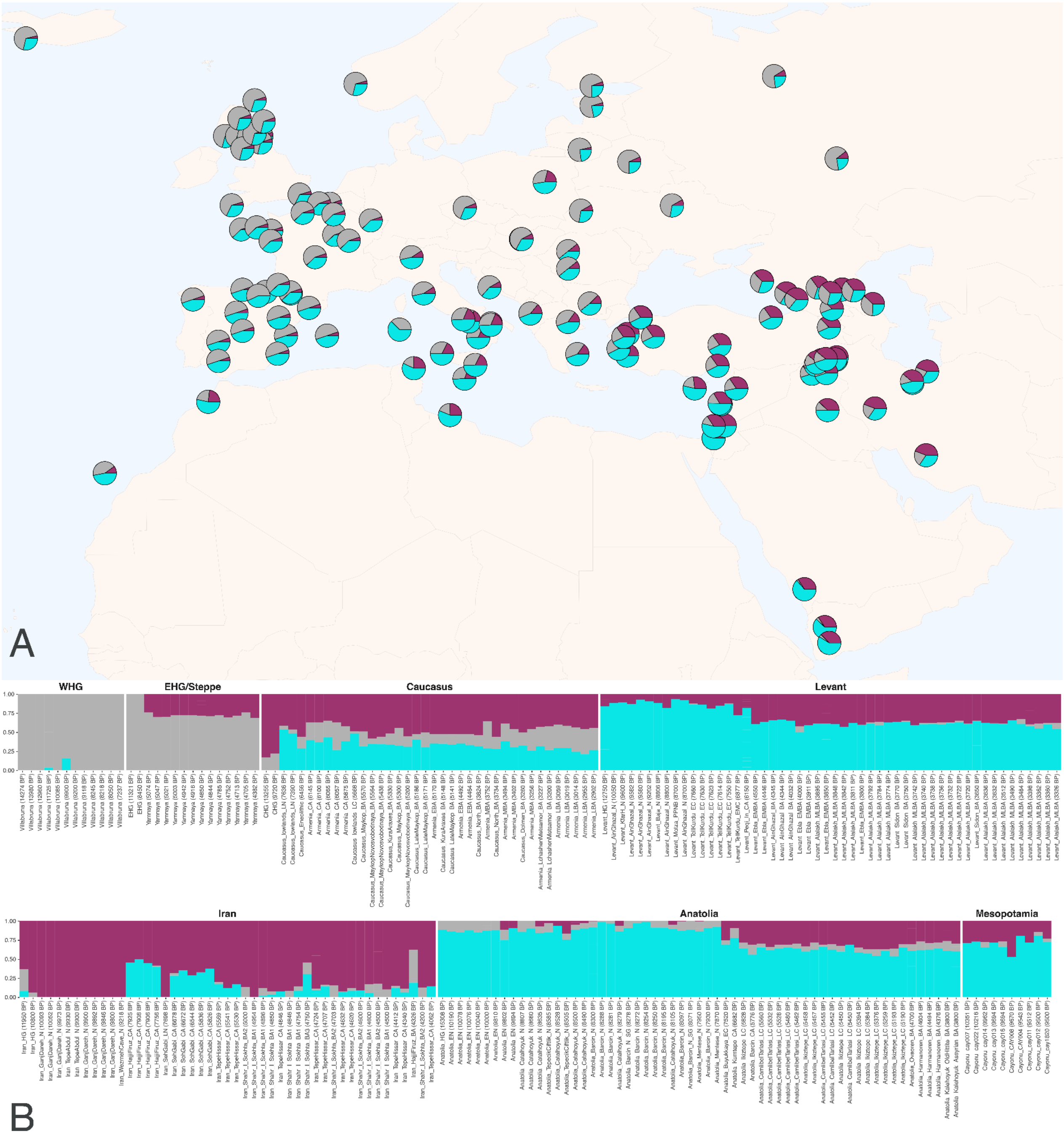

Fig. S6.
ADMIXTURE analysis of Southwest Asian ancient genomes at K=2,3,4,5. The x-axis was ordered temporally from earliest to latest.

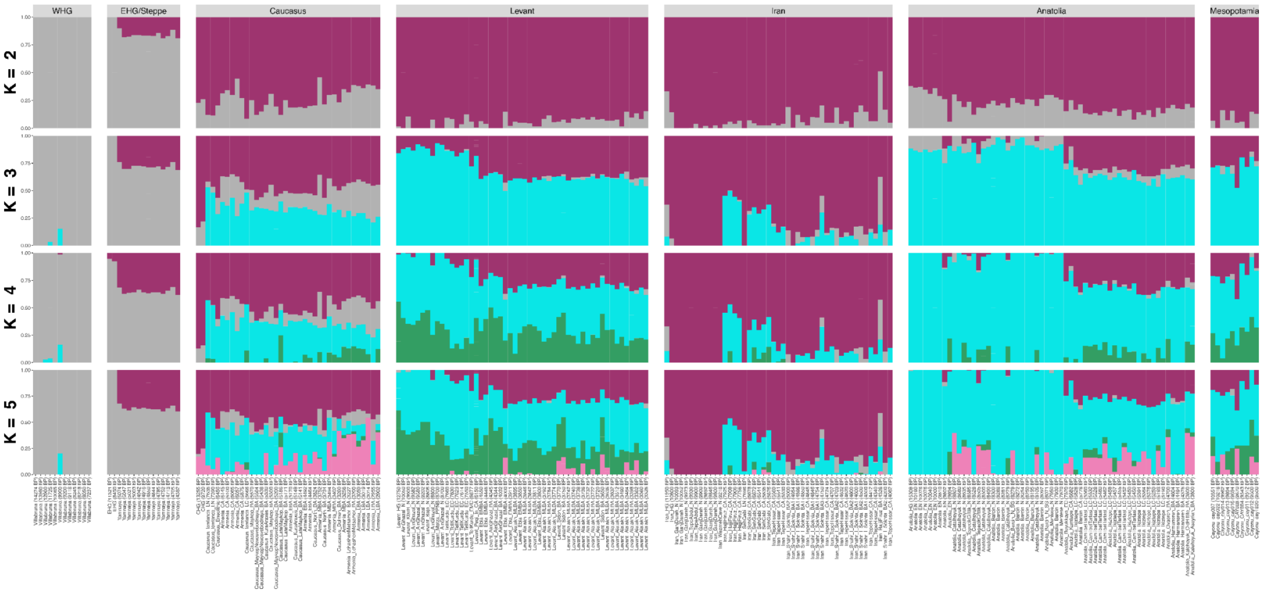

Fig. S7.
Modelling ancestry components of each individual Çayönü genome. Each bar represents a *qpAdm* model result, with grey bars indicating that the relevant models did not fit the data ($p < 0.01$). In panels **(A)** and **(B)**, the Anatolian Epipaleolithic genome from Pınarbaşı (Anatolia EP) and in panels **(C)** and **(D)**, Pre-Pottery Neolithic populations (Aşıklı and Boncuklu) from Central Anatolia were used as Anatolian sources. We modelled each individual as two- or three-way models shown in **A, C** and **B, D**, respectively. While in two-way models we used Anatolian and Central Zagros sources, three-way models additionally include South Levant Neolithic sources. Genomes are ordered on the y-axis from earliest (bottom) to latest (top). Individuals with >0.05X genome coverage are marked with asterisks. Notably, models with Levant-related components appear plausible only for these individuals with these relatively higher quality genomes in our sample. We did not include models of the cay008 genome, the results which are presented in Figure 2C.

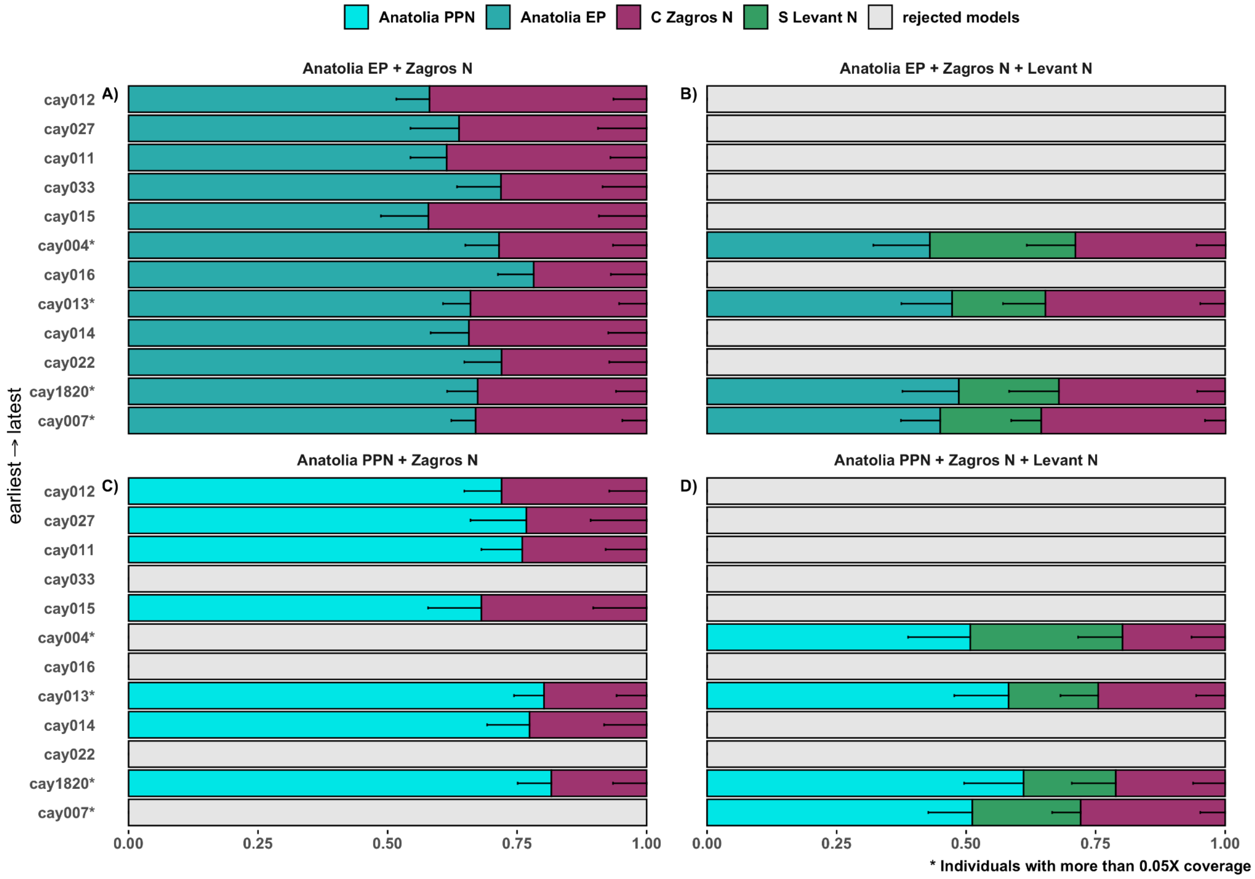

Fig. S8.

(A) Comparison of genetic diversity (1 - f_3) of co-buried individuals in Çayönü and in Central and West Anatolia. Small dots represent genetic distance between each pair, whereas larger dots show median values of each pair set. We observe a significant difference between genetic distances between co-burial pairs and pairs found in different buildings in Çayönü (permutation test p<0.001), and a similar but non-significant trend in Aşıklı (p>0.05). **(B)** Frequencies of co-buried individuals with or without identified close genetic kin.

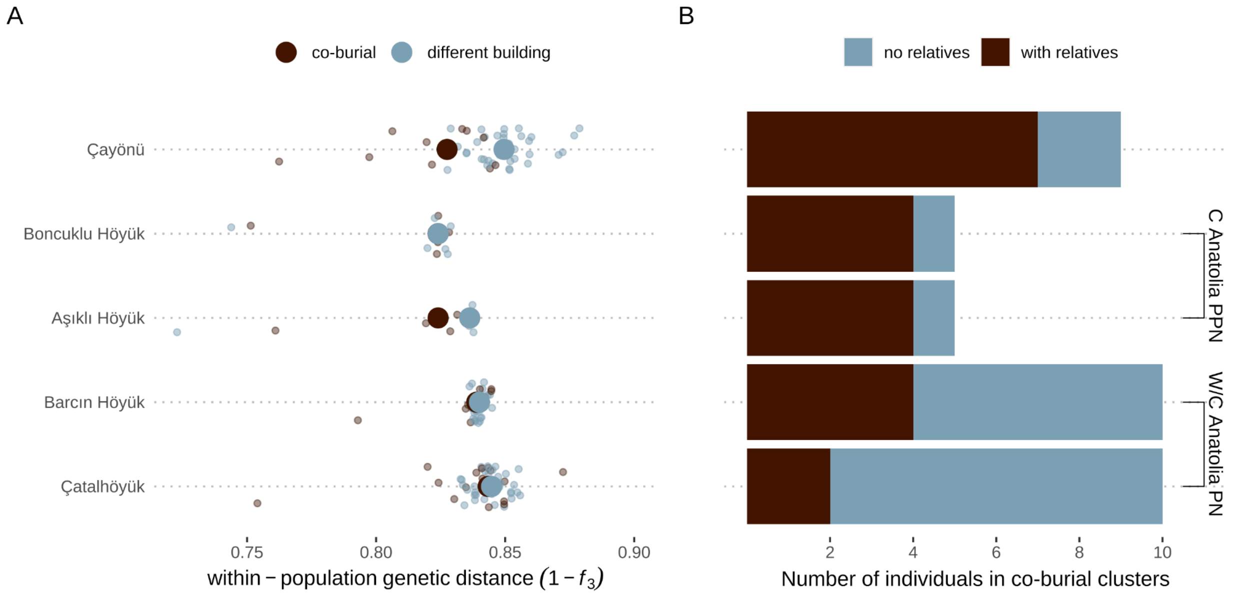

Fig. S9.
Temporal distribution of dated Çayönü individuals. **(A)** Summed Probability Distribution of radiocarbon dated individuals from all phases and individuals only from Cell Building structures. **(B)** Time differences in years between pairs of individuals buried in Cell Building structures, estimated by random sampling of the calibrated density curves of each pair 10,000 times. Colored dashed vertical lines show the mean of the pairwise time differences, black dotted lines show 0, and the horizontal bars show 95% quantiles estimated by random sampling. The filled points show significant shifts from 0 at 95% confidence, while empty points show non-significant results.

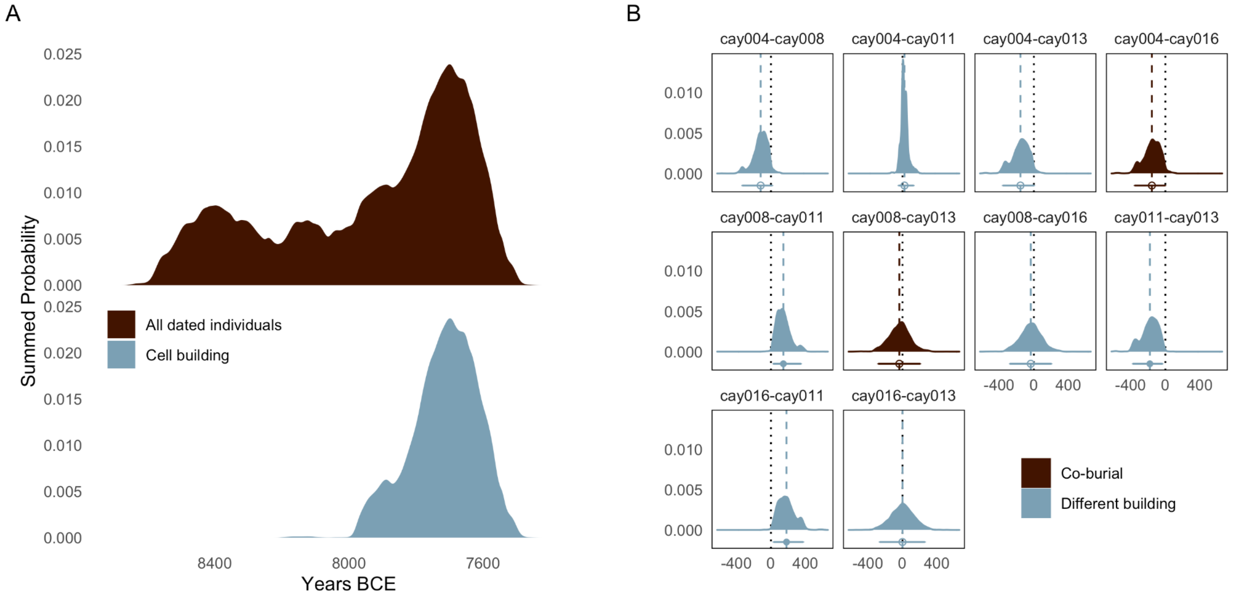

Fig. S10.

Simulated pedigrees for **(A)** paternal and **(B)** maternal relatives of cay008 to resolve her relationship with cay013 who is an adult female. Blue circles with dots correspond to the possible female third-degree relatives of cay008. Given that cay008 is an infant all relatives were simulated as related through the mother or father but not as descendants.

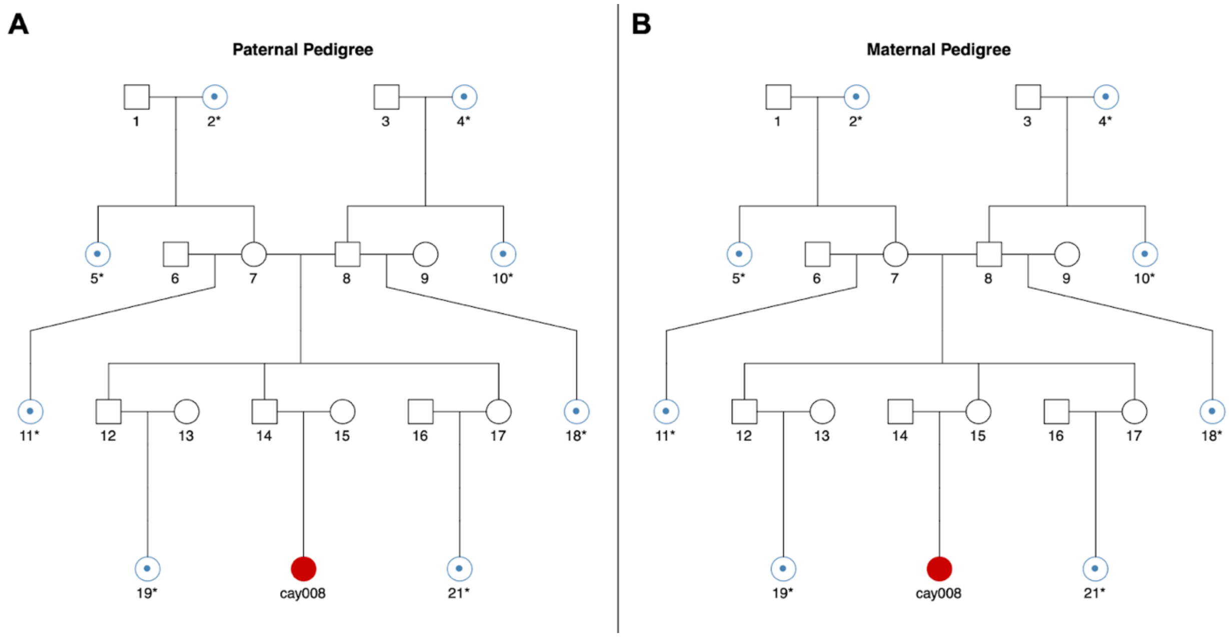

Fig. S11.
Theoretical kinship coefficient (θ) values between cay008 and her **(A)** paternal and **(B)** maternal relatives. Autosomal θ values are on the x-axes while X-chromosomal θ values are on the y-axes. The shaded area shows potential values similar to real data where X-chromosomal θ is higher than autosomal θ. Red dots represent θ values *1 - Normalised* P_0 between cay008 and cay013 individuals, whereas horizontal and vertical bars infer 95% confidence intervals. Triangles show the potential female relatives falling in this range. Annotated numbers correspond to relatives in the two pedigrees shown in Supplementary Figure 10. Jitter was added to the points to visualise all the data.

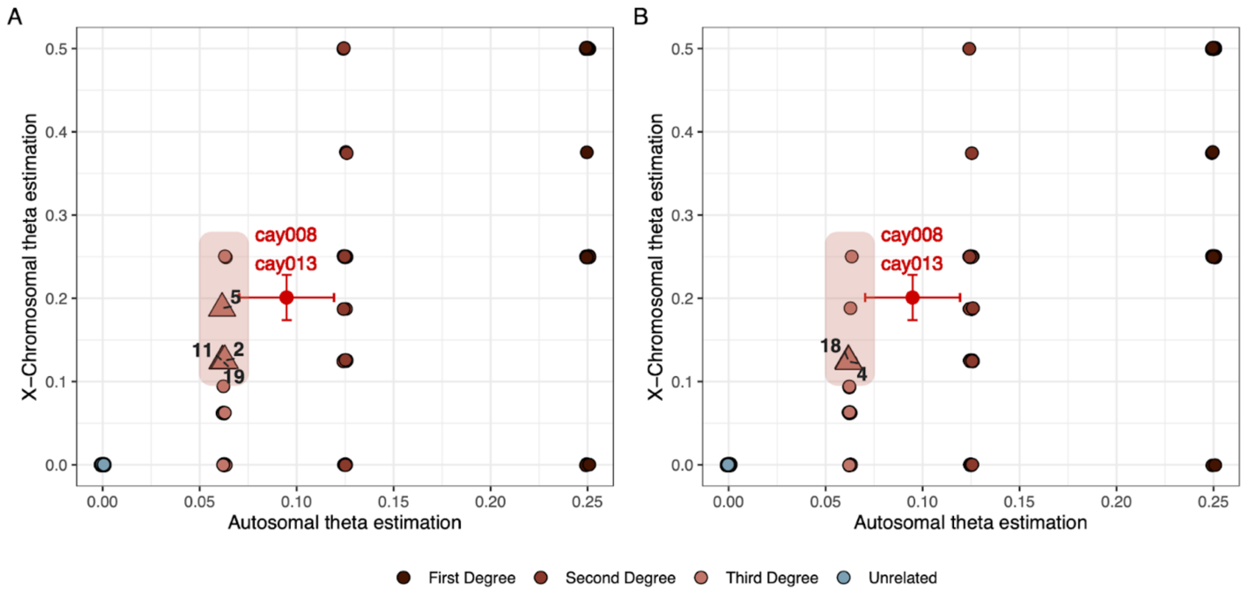

Fig. S12.
Direct computation of excess allele sharing between Çayönü and post-7000 BCE populations from Central/Western Anatolia calculated in the form of *D*(*test*, *reference*; CHG, Çayönü) in which *test* corresponds to Pottery Neolithic and later populations from Anatolia and *reference* corresponds to Epipaleolithic (Pınarbaşı) and Pre-Pottery Neolithic populations (Aşıklı and Boncuklu). Empty dots show insignificant results and vertical bars represent ±2 standard errors.

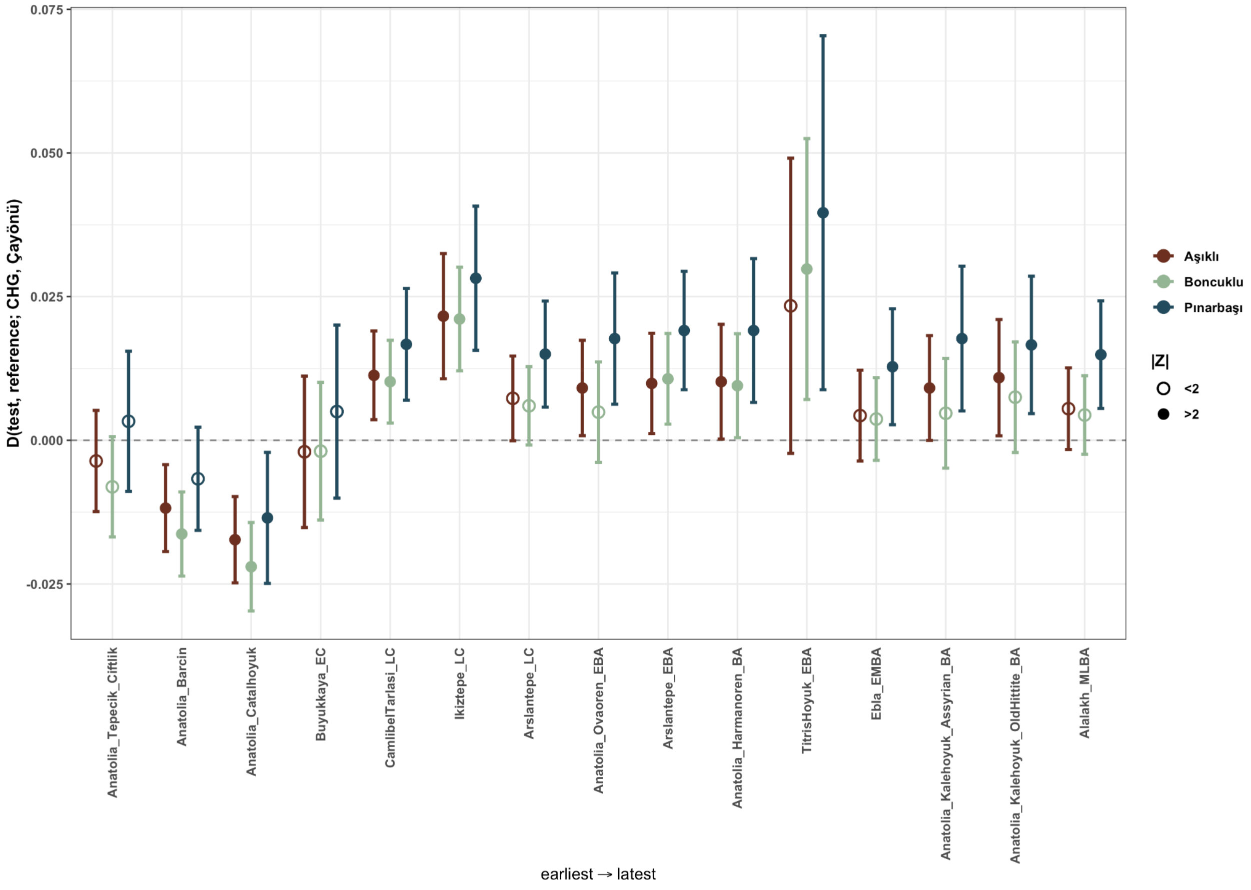

Fig. S13.
Phylogenetic tree of Y-chromosome computed with *PathPhynder* (*80*). All male individuals were placed onto the tree using the *best path* method.

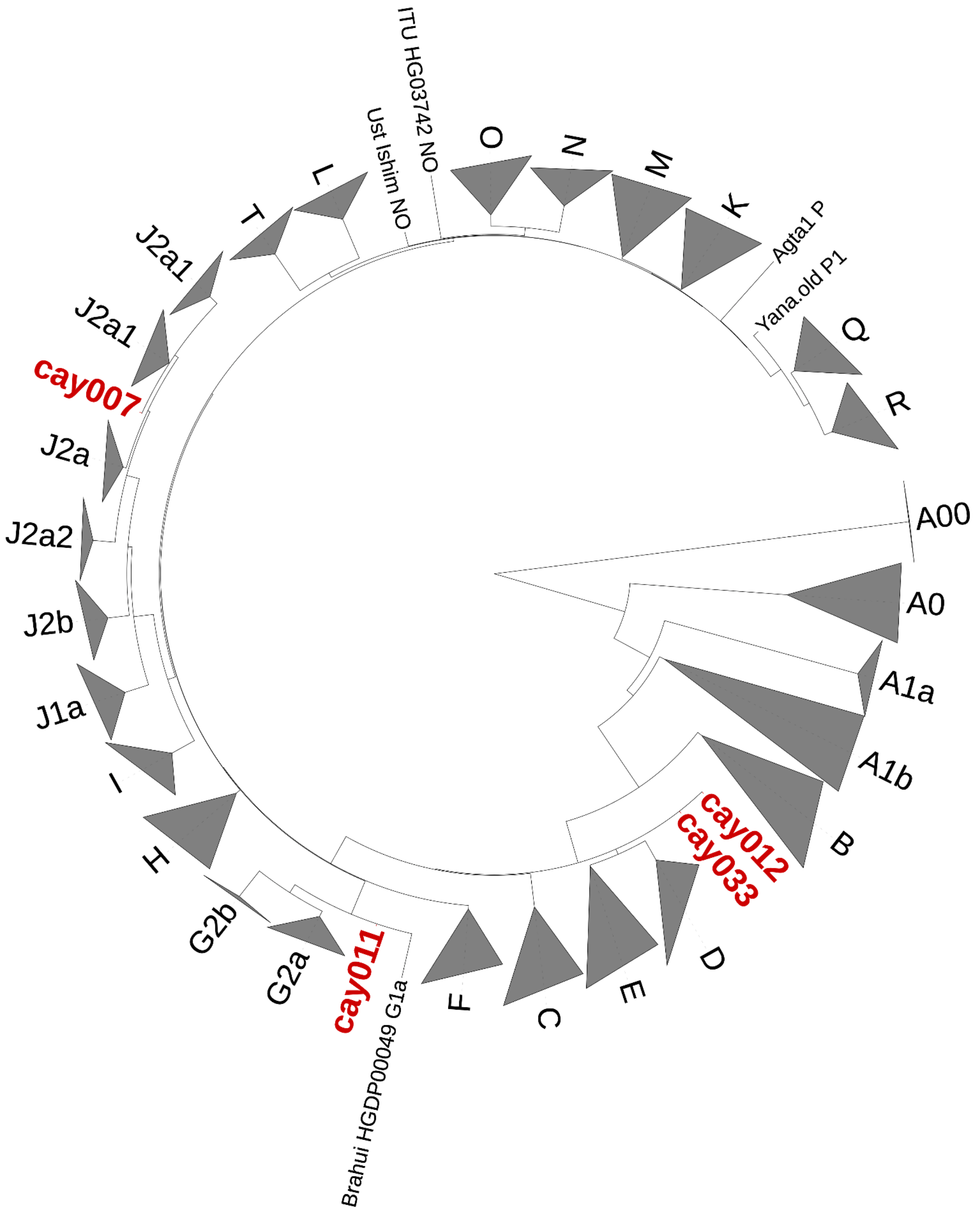

Table S1.

Archaeological contexts and anthropological characteristics of all screened Çayönü individuals.

**Building**	**Sub-phase**	**DNA lab No**	**DNA Preservation**	**Skeleton Number**	**Genetic Sex**	**Age at Death**	**Burial Type**	**Basic Pathologies**
CA	c1 (PPNB)	cay001	-	ÇT'86 S.1	Male	40-45 years old (Middle Adult)	Tightly flexed, NNE-SSW oriented, lying R, face down	Slightly developed osteoarthritis (OA) on the hip and shoulder joints; moderate arthritic changes in thoracic and lumbar vertebrae and knee joint. New bone formation on the internal table of the cranium and pelvic bones.
CA	c1 (PPNB)	cay002	-	ÇT'78 S.12	Unknown	11-12 years old (Child)	Tightly flexed, primary, NE-SW oriented, lying R, face down	A healed depressed trauma on the right parietal bone; moderate cribra orbitalia on the right orbital roof.
CXa	c3 or c1 (PPNB)	cay003	-	ÇT'72 S.10a	Female	35-45 years old (Middle Adult)	Flexed, primary, W-E oriented, lying L, face S	A healed fracture on left big toe.
CL	c1(PPNB)	cay004	+	ÇT'78 S.16	Female	33-46 years old (Middle adult)	Partially flexed W-E oriented, lying L, face SW	Healed fracture on the distal end of left ulnae; Monteggia fracture on right ulnae; periostitis on fibulae, slightly porotic hyperostosis on the cranium; slightly developed osteoarthritis on proximal end ulnae; Schmorl's nodule on 5th lumbar vertebrae.
CR	c3 (PPNB)	cay005	-	ÇT'78 S.9	Male	Adult (>15)	It was excavated between walls	Three healed depressed fractures (one of them is on the midline of the frontal bone and two of them are left parietal bone; slightly developed PH; severe osteoarthritic changes on the cervical vertebrae.

CXa	c1(PPNB)	cay00 6	-	ÇT’81 S.6	Unknown	4-5 years old (Child)	-	-
GE	Mid g (PPNA-PPNB transition)	cay00 7	+	ÇT’81 S.2a	Male	20-23 years old (Young adult)	Flexed, NW-SE oriented, lying R	A healed depressed trauma on right parietal bone and a healed fracture on felt 5th phalanges of the foot; slightly developed osteoarthritis on the right foot bones.
CA	c1 (PPNB)	cay00 8	+	ÇT’86 S.2	Female	1-2 years old (Infant)	Flexed? skull in W	Cauterization; circular type intentional head-shaping with post-coronal depression; woven bone formation on inner surface of the occipital bone.
CXa	c3 or c1 (PPNB)	cay00 9	-	ÇT’72 S.8b	Female	2-4 years old (Child)	Unknown, represented by teeth (she was found with ÇT’72 S.8a)	-
CN	c1 (PPNB)	cay01 0	-	ÇT’78 S.2	Unknown	2-2,5 years old (Infant)	Flexed	Severe hypoplasia; Possible head-shaping with the sign of post-coronal depression.
CN	c1 (PPNB)	cay01 1	+	ÇT’78 S.7	Male	33-42 years old (Middle adult)	Flexed, primary, SSE-NNW oriented, lying L, face down	Two healed depressed traumas (one of them is on left parietal and the other is on the frontal); healed periostitis on tibiae; Healed fracture on the left big toe, slightly osteoarthritis on the right ulnae and severe on cervical vertebrae.
CN	c1 (PPNB)	cay01 2	+	ÇT’78 S.6	Male	Nearly 12 years old (Child)	Tightly flexed, primary, SE-NW oriented, lying L, face down	Possible Scurvy? New bone formation is observed around the muscular attachment of all long bones and porosity on the ramus of the mandible.
CA	c1 (PPNB)	cay01 3	+	ÇT’78 S.21	Female	43-58 years old (Old adult)	Tightly flexed, NNE-SSW oriented, lying R, face down	Depressed trauma on the occipital; A fracture left costa, and periostitis on this fragment; slightly developed porotic hyperostosis and cribra orbitalia; slight osteoarthritis on the

								hand and foot phalanges; A possible head-shaping with a plano-occipital flattening.
CL	c1(PPNB)	cay01 4	+		Female	Adult	-	-
CL	c1(PPNB)	cay01 5	+	ÇT'81 S.15	Female	40-45 years old (Middle adult)	Flexed, primary, SSW-N oriented, lying R, face W	A healed Colles' fracture on the left radius; a healed fracture on medial foot phalange; periostitis on sacrum, slight porotic hyperostosis and cribra orbitalia; slight osteoarthritis on the distal radius, thoracic and lumbar vertebrae.
CL	c1(PPNB)	cay01 6	+	ÇT'81 S.8	Female	40-55 years old (Old adult)	Tightly flexed, S-N oriented, lying on the R	A healed Colles' fracture on the left radius; moderate porotic hyperostosis and cribra orbitalia; severe osteoarthritis on the left wrist bones, slightly on the right shoulder, moderate on the thoracic and severe on the lumbar vertebrae.
CV (CXa)	c3 or c1 (PPNB)	cay01 7	-	ÇT'70 S.3	Female	28-37 years (Middle Adult)	Tightly flexed, S-N oriented, lying R, face SW	Cribra orbitalia on the left orbital roof.
GB(b)	g (upper) (early PPNB)	cay01 8	+	ÇT'70 S.13	Female	3-9 months (Infant)	Flexed, partly twisted, primary, SW-ENE oriented, lying R	No pathology observed.
GB(b)	g (upper) (early PPNB)	cay01 9	-	ÇT'70 S.10a	Female	Adult (>15)	Flexed, primary, ENE-WSW oriented, lying R	Healed depressed trauma on the R parietal bone; Hyperostosis frontalis interna; Disease idiopathic skeletal hyperostosis and intentional head-shaping with a post-coronal depression.

GB(b)	g (upper) (early PPNB)	cay020	+	ÇT'70 S.11b	Female	3-9 months (Infant)	Flexed, primary, lying R	Severe PH on parietal; infection on internal surface of occipital.
CXa	c1(PPNB)	cay021	-	ÇT'72 S.9a	Female	Adult (>15)	Flexed, W-E oriented, lying R	Healed depressed trauma on the bregma region.
CR	c3 or cp3 (PPNB)	cay022	+	ÇT'78 S.25	Female	9-12 months (Infant)	-	New bone formation on the internal table of the cranium; moderate porotic hyperostosis.
CA	c1 (PPNB)	cay023	-	ÇT'72 S.5	Female	8-8,5 years (Child)	Flexed, secondary, NE-SW oriented, lying R, face E	Slight cribra orbitalia in both orbital roofs.
CA	c1 (PPNB)	cay024	-	ÇT'78 S.20	Female	Middle adult	Tightly flexed, SE-NW orientation, lying L ?, face down	Slight porotic hyperostosis and cribra orbitalia. Osteoarthritic changes on cervical vertebrae.
CA	c1 (PPNB)	cay025	-	ÇT'78 S.13	Male	Adult (>15)	Tightly flexed, NNW-SSE oriented, lying R, face down	Healed fracture on the right 2nd and 3rd metacarpal and distal phalanges, slight osteoarthritis on the right distal radius.
21N	c2 (PPNB)	cay026	-	ÇT'78 S.28b	Female	Old adult	-	Two healed depression traumas on the cranium (one is on the left frontal tuber and other is on the occipital; hyperostosis frontalis interna; slight osteoarthritis on right shoulder; moderate on the cervical vertebrae.
CN	c1(PPNB)	cay027	+	ÇT'78 S.1	Female	Adult (>15)	Flexed? secondary? SW-NE oriented, face?	Infection on the right tibia.
CL	c1 (PPNB)	cay028	-	ÇT'78 S.17	Female	22-24 years (Young adult)	Semi flexed, N-S directed lying on right side and partially on stomach	Slight porotic hyperostosis.

CA	c1 (PPNB)	cay029	-	ÇT'78 S.19	Female	40-45 years (Middle adult)	Tightly flexed, SSW-NNE oriented, lying on the left side	Healed depressed trauma on the skull, maxillary sinusitis; periostitis on the right tibia; slight osteoarthritis on cervical vertebrae.
CXa	c1 (PPNB)	cay030	-	ÇT'81 S.7	Female	Adult (>15)	Flexed	Healed depressed trauma on the right parietal.
CXa	c1 (PPNB)	cay031	-	ÇT'81 S. 4	Male	Adult (>15)	Flexed	Two healed depressed traumas on the right parietal bone and slight porotic hyperostosis.
GE	Mid g (PPNA-PPNB transition)	cay032	-	ÇT'81 S.2b	Male	Young adult	Together with 81 S.2a	Two healed fractures on the right foot bones; periostitis on the right tibia.
CL	c1 (PPNB)	cay033	+	ÇT'84 S.60	Male	8-8,5 years (Child)	Under 81 S.15 (cay016)	Hematoma on the left femoral diaphysis.

Additional Supplementary Tables

Table S2. Material culture elements from PPN occupations at given sites.
Table S3. Sequencing statistics of Çayönü individuals. Green rows represent merged libraries of each individual.
Table S4. Published ancient and modern genomes used in this study.
Table S5. D-statistics showing population affinities in Southwest Asia.
Table S6. Two- and three-way qpAdm models.
Table S7. Permutation test results of with-in population diversity in Southwest Asia. Upper triangle shows p-values and bottom triangle shows the effect size.
Table S8. Kinship analyses on autosomes performed using READ and NGSRelate v2.
Table S9. Kinship analyses on X-chromosome performed using READ and NGSRelate v2.

REFERENCES AND NOTES

1. J. Notroff, O. Dietrich, K. Schmidt, in *Approaching Monumentality in Archaeology*, J. Osborne, Ed. (State University of New York Press, 2014), pp. 83–105.

2. C. Kabukcu, E. Asouti, N. Pöllath, J. Peters, N. Karul, Pathways to plant domestication in Southeast Anatolia based on new data from aceramic Neolithic Gusir Höyük. *Sci. Rep.* **11**, 1–15 (2021).

3. J. Peters, N. Pöllath, B. S. Arbuckle, "The emergence of livestock husbandry in Early Neolithic Anatolia" in *The Oxford Handbook of Zooarchaeology*, U. Albarella, M. Rizzetto, H. Russ, K. Vickers, S. Viner-Daniels, Eds. (Oxford University Press, 2017), vol. 1, pp. 247–265.

4. J. Peters, K. Schmidt, L. Dietrich, O. Dietrich, N. Pöllath, M. Kinzel, L. Clare, Göbekli Tepe: Agriculture and domestication. *Encycl. Global Archaeol*, 3065–3068 (2019).

5. B. S. Arbuckle, S. W. Kansa, E. Kansa, D. Orton, C. Çakirlar, L. Gourichon, L. Atici, A. Galik, A. Marciniak, J. Mulville, H. Buitenhuis, D. Carruthers, B. de Cupere, A. Demirergi, S. Frame, D. Helmer, L. Martin, J. Peters, N. Pöllath, K. Pawłowska, N. Russell, K. Twiss, D. Würtenberger, Data sharing reveals complexity in the westward spread of domestic animals across neolithic Turkey. *PLOS ONE* **9**, e99845 (2014).

6. E. Weiss, D. Zohary, The Neolithic Southwest Asian founder crops their biology and archaeobotany. *Curr. Anthropol.* **52**, S237–S254 (2011).

7. M. Feldman, E. Fernández-Domínguez, L. Reynolds, D. Baird, J. Pearson, I. Hershkovitz, H. May, N. Goring-Morris, M. Benz, J. Gresky, R. A. Bianco, A. Fairbairn, G. Mustafaoğlu, P. W. Stockhammer, C. Posth, W. Haak, C. Jeong, J. Krause, Late Pleistocene human genome suggests a local origin for the first farmers of central Anatolia. *Nat. Commun.* **10**, 1–10 (2019).

8. M. Gallego-Llorente, S. Connell, E. R. Jones, D. C. Merrett, Y. Jeon, A. Eriksson, V. Siska, C. Gamba, C. Meiklejohn, R. Beyer, S. Jeon, Y. S. Cho, M. Hofreiter, J. Bhak, A. Manica, R. Pinhasi, The genetics of an early Neolithic pastoralist from the Zagros, Iran. *Sci. Rep.* **6**, 1–7 (2016).

9. G. M. Kılınç, A. Omrak, F. Özer, T. Günther, A. M. Büyükkarakaya, E. Bıçakçı, D. Baird, H. M. Dönertaş, A. Ghalichi, R. Yaka, D. Koptekin, S. C. Açan, P. Parvizi, M. Krzewińska, E. A. Daskalaki, E. Yüncü, N. D. Dağtaş, A. Fairbairn, J. Pearson, G. Mustafaoğlu, Y. S. Erdal, Y. G. Çakan, İ. Togan, M. Somel, J. Storå, M. Jakobsson, A. Götherström, The demographic development of the first farmers in Anatolia. *Curr. Biol.* **26**, 2659–2666 (2016).

10. G. M. Kılınç, D. Koptekin, Ç. Atakuman, A. P. Sümer, H. M. Dönertaş, R. Yaka, C. C. Bilgin, A. M. Büyükkarakaya, D. Baird, E. Altınışık, P. Flegontov, A. Götherström, İ. Togan, M. Somel, Archaeogenomic analysis of the first steps of Neolithization in Anatolia and the Aegean. *Proc. Biol. Sci.* **284**, 20172064 (2017).

11. I. Lazaridis, N. Patterson, A. Mittnik, G. Renaud, S. Mallick, K. Kirsanow, P. H. Sudmant, J. G. Schraiber, S. Castellano, M. Lipson, B. Berger, C. Economou, R. Bollongino, Q. Fu, K. I. Bos, S. Nordenfelt, H. Li, C. De Filippo, K. Prüfer, S. Sawyer, C. Posth, W. Haak, F. Hallgren, E. Fornander, N. Rohland, D. Delsate, M. Francken, J. M. Guinet, J. Wahl, G. Ayodo, H. A. Babiker, G. Bailliet, E. Balanovska, O. Balanovsky, R. Barrantes, G. Bedoya, H. Ben-Ami, J. Bene, F. Berrada, C. M. Bravi, F. Brisighelli, G. B. J. Busby, F. Cali, M. Churnosov, D. E. C. Cole, D. Corach, L. Damba, G. Van Driem, S. Dryomov, J. M. Dugoujon, S. A. Fedorova, I. G. Romero, M. Gubina, M. Hammer, B. M. Henn, T. Hervig, U. Hodoglugil, A. R. Jha, S. Karachanak-Yankova, R. Khusainova, E. Khusnutdinova, R. Kittles, T. Kivisild, W. Klitz, V. Kučinskas, A. Kushniarevich, L. Laredj, S. Litvinov, T. Loukidis, R. W. Mahley, B. Melegh, E. Metspalu, J. Molina, J. Mountain, K. Näkkäläjärvi, D. Nesheva, T. Nyambo, L. Osipova, J. Parik, F. Platonov, O. Posukh, V. Romano, F. Rothhammer, I. Rudan, R. Ruizbakiev, H. Sahakyan, A. Sajantila, A. Salas, E. B. Starikovskaya, A. Tarekegn, D. Toncheva, S. Turdikulova, I. Uktveryte, O. Utevska, R. Vasquez, M. Villena, M. Voevoda, C. A. Winkler, L. Yepiskoposyan, P. Zalloua, T. Zemunik, A. Cooper, C. Capelli, M. G. Thomas, A. Ruiz-Linares, S. A. Tishkoff, L. Singh, K. Thangaraj, R. Villems, D. Comas, R. Sukernik, M. Metspalu, M. Meyer, E. E. Eichler, J. Burger, M. Slatkin, S. Pääbo, J. Kelso, D. Reich, J. Krause, Ancient human genomes suggest three ancestral populations for present-day Europeans. *Nature* **513**, 409–413 (2014).

12. I. Lazaridis, D. Nadel, G. Rollefson, D. C. Merrett, N. Rohland, S. Mallick, D. Fernandes, M. Novak, B. Gamarra, K. Sirak, S. Connell, K. Stewardson, E. Harney, Q. Fu, G. Gonzalez-Fortes, E. R. Jones, S. A. Roodenberg, G. Lengyel, F. Bocquentin, B. Gasparian, J. M. Monge, M. Gregg, V. Eshed, A. S. A.-S. Mizrahi, C. Meiklejohn, F. Gerritsen, L. Bejenaru, M. Blüher, A. Campbell, G. Cavalleri, D. Comas, P. Froguel, E. Gilbert, S. M. Kerr, P. Kovacs, J. Krause, D. McGettigan, M. Merrigan, D. A. Merriwether, S. O'Reilly, M. B. Richards, O. Semino, M. Shamoon-Pour, G. Stefanescu, M. Stumvoll, A. Tönjes, A. Torroni, J. F. Wilson, L. Yengo, N. A. Hovhannisyan, N. Patterson, R. Pinhasi, D. Reich, S. O'Reilly, M. B. Richards, O. Semino, M. Shamoon-Pour, G. Stefanescu, M. Stumvoll, A. Tönjes, A. Torroni, J. F. Wilson, L. Yengo, N. A. Hovhannisyan, N. Patterson, R. Pinhasi, D. Reich, Genomic insights into the origin of farming in the ancient Near East. *Nature* **536**, 419–424 (2016).

13. R. Yaka, I. Mapelli, D. Kaptan, A. Doğu, M. Chyleński, Ö. D. Erdal, D. Koptekin, K. B. Vural, A. Bayliss, C. Mazzucato, E. Fer, S. S. Çokoğlu, V. K. Lagerholm, M. Krzewińska, C. Karamurat, H. C. Gemici, A. Sevkar, N. D. Dağtaş, G. M. Kılınç, D. Adams, A. R. Munters, E. Sağlıcan, M. Milella, E. M. J. Schotsmans, E. Yurtman, M. Çetin, S. Yorulmaz, N. E. Altınışık, A. Ghalichi, A. Juras, C. C. Bilgin, T. Günther, J. Storå, M. Jakobsson, M. de Kleijn, G. Mustafaoğlu, A. Fairbairn, J. Pearson, İ. Togan, N. Kayacan, A. Marciniak, C. S. Larsen, I. Hodder, Ç. Atakuman, M. Pilloud, E. Sürer, F. Gerritsen, R. Özbal, D. Baird, Y. S. Erdal, G. Duru, M. Özbaşaran, S. D. Haddow, C. J. Knüsel, A. Götherström, F. Özer, M. Somel, Variable kinship patterns in Neolithic Anatolia revealed by ancient genomes. *Curr. Biol.* **31**, 2455–2468.e18 (2021).

14. E. Skourtanioti, Y. S. Erdal, M. Frangipane, F. B. Restelli, K. A. Yener, F. Pinnock, P. Matthiae, R. Özbal, U.-D. Schoop, F. Guliyev, T. Akhundov, B. Lyonnet, E. L. Hammer, S. E. Nugent, M. Burri, G. U. Neumann, S. Penske, T. Ingman, M. Akar, R. Shafiq, G. Palumbi, S. Eisenmann, M. D'Andrea, A. B. Rohrlach, C. Warinner, C. Jeong, P. W. Stockhammer, W. Haak, J. Krause, Genomic history of neolithic to bronze age Anatolia, Northern Levant, and Southern Caucasus. *Cell* **181**, 1158–1175.e28 (2020).

15. E. Fernández, A. Pérez-Pérez, C. Gamba, E. Prats, P. Cuesta, J. Anfruns, M. Molist, E. Arroyo-Pardo, D. Turbón, Ancient DNA analysis of 8000 B.C. Near Eastern farmers supports an early

neolithic pioneer maritime colonization of Mainland Europe through cyprus and the Aegean Islands. *PLOS Genet.* **10**, e1004401 (2014).

16. A. Erim-Özdoğan, Çayönü, in *The Neolithic In Turkey New Excavations & New Research* (Arkeoloji ve Sanat Yayınları, 2011), pp. 185–269.

17. W. van Zeist, G. J. de Roller, The plant husbandry of aceramic Çayönü, SE Turkey. *Palaeohistoria*, 65–96 (1994).

18. H. Hongo, J. Pearson, B. Öksüz, G. Ilgezdi, The process of ungulate domestication at Çayönü, Southeastern Turkey: A multidisciplinary approach focusing on Bos sp. and cervus elaphus. *Anthropozoologica* **44**, 63–78 (2009).

19. M. T. C. Affonso, E. Pernicka, in *Lux Orientis. Archäologie zwischen Asien und Europa, Festschrift für Harald Hauptmann*, R. M. Boehmer, J. Maran, Eds. (Leidorf, 2001), vol. 65, pp. 9–13.

20. M. Özdoğan, A. Özdoğan, in *The Beginnings of Metallurgy*, A. Hauptmann, E. Pernicka, T. Rehren, Ü. Yalçın, Eds. (Der Anschnitt, 1999), pp. 13–22.

21. M. Rosenberg, A. Erim-Özdoğan, "The Neolithic in Southeastern Anatolia" in *The Oxford Handbook of Ancient Anatolia: (10,000-323 BCE)*, G. McMahon, S. Steadman, Eds. (Oxford University Press, 2012).

22. F. Broushaki, M. G. Thomas, V. Link, S. López, L. van Dorp, K. Kirsanow, Z. Hofmanová, Y. Diekmann, L. M. Cassidy, D. Díez-del-Molino, A. Kousathanas, C. Sell, H. K. Robson, R. Martiniano, J. Blöcher, A. Scheu, S. Kreutzer, R. Bollongino, D. Bobo, H. Davoudi, O. Munoz, M. Currat, K. Abdi, F. Biglari, O. E. Craig, D. G. Bradley, S. Shennan, K. R. Veeramah, M. Mashkour, D. Wegmann, G. Hellenthal, J. Burger, Early Neolithic genomes from the eastern Fertile Crescent. *Science* **353**, 499–503 (2016).

23. Z. Hofmanová, S. Kreutzer, G. Hellenthal, C. Sell, Y. Diekmann, D. Díez-del-Molino, L. van Dorp, S. López, A. Kousathanas, V. Link, K. Kirsanow, L. M. Cassidy, R. Martiniano, M. Strobel, A. Scheu, K. Kotsakis, P. Halstead, S. Triantaphyllou, N. Kyparissi-Apostolika, D.

Urem-Kotsou, C. Ziota, F. Adaktylou, S. Gopalan, D. M. Bobo, L. Winkelbach, J. Blöcher, M. Unterländer, C. Leuenberger, Ç. Çilingiroğlu, B. Horejs, F. Gerritsen, S. J. Shennan, D. G. Bradley, M. Currat, K. R. Veeramah, D. Wegmann, M. G. Thomas, C. Papageorgopoulou, J. Burger, Early farmers from across Europe directly descended from Neolithic Aegeans. *Proc. Natl. Acad. Sci. U.S.A.* **113**, 6886–6891 (2016).

24. E. R. Jones, G. Gonzalez-Fortes, S. Connell, V. Siska, A. Eriksson, R. Martiniano, R. L. McLaughlin, M. Gallego Llorente, L. M. Cassidy, C. Gamba, T. Meshveliani, O. Bar-Yosef, W. Müller, A. Belfer-Cohen, Z. Matskevich, N. Jakeli, T. F. G. Higham, M. Currat, D. Lordkipanidze, M. Hofreiter, A. Manica, R. Pinhasi, D. G. Bradley, Upper Palaeolithic genomes reveal deep roots of modern Eurasians. *Nat. Commun.* **6**, 8912 (2015).

25. I. Mathieson, I. Lazaridis, N. Rohland, S. Mallick, N. Patterson, S. A. Roodenberg, E. Harney, K. Stewardson, D. Fernandes, M. Novak, K. Sirak, C. Gamba, E. R. Jones, B. Llamas, S. Dryomov, J. Pickrell, J. L. Arsuaga, J. M. B. De Castro, E. Carbonell, F. Gerritsen, A. Khokhlov, P. Kuznetsov, M. Lozano, H. Meller, O. Mochalov, V. Moiseyev, M. A. R. R. Guerra, J. Roodenberg, J. M. Vergès, J. Krause, A. Cooper, K. W. Alt, D. Brown, D. Anthony, C. Lalueza-Fox, W. Haak, R. Pinhasi, D. Reich, Genome-wide patterns of selection in 230 ancient Eurasians. *Nature* **528**, 499–503 (2015).

26. N. Patterson, P. Moorjani, Y. Luo, S. Mallick, N. Rohland, Y. Zhan, T. Genschoreck, T. Webster, D. Reich, Ancient admixture in human history. *Genetics* **192**, 1065–1093 (2012).

27. B. M. Peter, D. Petkova, J. Novembre, Genetic landscapes reveal how human genetic diversity aligns with geography. *Mol. Biol. Evol.* **37**, 943–951 (2020).

28. L. J. L. Handley, A. Manica, J. Goudet, F. Balloux, Going the distance: Human population genetics in a clinal world. *Trends Genet.* **23**, 432–439 (2007).

29. D. Petkova, J. Novembre, M. Stephens, Visualizing spatial population structure with estimated effective migration surfaces. *Nat. Genet.* **48**, 94–100 (2016).

30. O. Barge, C. Chataigner, Diffusion of Anatolian and Caucasian obsidian in the Zagros Mountains and the highlands of Iran: Elements of explanation in "least cost path" models. *Quat. Int.* **467**, 297–322 (2018).

31. E. Asouti, D. Baird, C. Kabukcu, K. Swinson, L. Martin, A. Garcia-Suarez, E. Jenkins, K. Rasheed, The Zagros Epipalaeolithic revisited: New excavations and 14C dates from Palegawra cave in Iraqi Kurdistan. *PLOS ONE* **15**, e0239564 (2020).

32. D. Baird, E. Asouti, L. Astruc, A. Baysal, E. Baysal, D. Carruthers, A. Fairbairn, C. Kabukcu, E. Jenkins, K. Lorentz, C. Middleton, J. Pearson, A. Pirie, Juniper smoke, skulls and wolves' tails. The Epipalaeolithic of the Anatolian plateau in its South-west Asian context; insights from Pınarbaşı. *Levant* **45**, 175–209 (2013).

33. N. Marchi, L. Winkelbach, I. Schulz, M. Brami, Z. Hofmanová, J. Blöcher, C. S. Reyna-Blanco, Y. Diekmann, A. Thiéry, A. Kapopoulou, V. Link, V. Piuz, S. Kreutzer, S. M. Figarska, E. Ganiatsou, A. Pukaj, T. J. Struck, R. N. Gutenkunst, N. Karul, F. Gerritsen, J. Pechtl, J. Peters, A. Zeeb-Lanz, E. Lenneis, M. Teschler-Nicola, S. Triantaphyllou, S. Stefanović, C. Papageorgopoulou, D. Wegmann, J. Burger, L. Excoffier, The genomic origins of the world's first farmers. *Cell* **185**, 1842–1859.e18 (2022).

34. R. Matthews, A. Richardson, O. Maeda, in *The Early Neolithic of the Eastern Fertile Crescent: Excavations at Bestansur and Shimshara, Iraqi Kurdistan* (Oxbow Books, 2020), vol. 2, pp. 461–533.

35. A. Richardson, in *The Early Neolithic of the Eastern Fertile Crescent: Excavations at Bestansur and Shimshara, Iraqi Kurdistan* (Oxbow Books, 2020), vol. 2, pp. 533–567.

36. H. Ringbauer, J. Novembre, M. Steinrücken, Parental relatedness through time revealed by runs of homozygosity in ancient DNA. *Nat. Commun.* **12**, 1–11 (2021).

37. F. C. Ceballos, K. Gürün, N. E. Altınışık, H. C. Gemici, C. Karamurat, D. Koptekin, K. B. Vural, I. Mapelli, E. Sağlıcan, E. Sürer, Y. S. Erdal, A. Götherström, F. Özer, Ç. Atakuman, M.

Somel, Human inbreeding has decreased in time through the Holocene. *Curr. Biol.* **31**, 3925–3934.e8 (2021).

38. A. Palmisano, D. Lawrence, M. W. de Gruchy, A. Bevan, S. Shennan, Holocene regional population dynamics and climatic trends in the Near East: A first comparison using archaeo-demographic proxies. *Quat. Sci. Rev.* **252**, 106739 (2021).

39. R. J. Braidwood, L. Braidwood, Jarmo: A village early farmers in Iraq. *Antiquity* **24**, 189–195 (1950).

40. R. J. Braidwood, The agricultural revolution. *Sci. Am.* **203**, 131–148 (1960).

41. H. Çambel, R. J. Braidwood, An early farming village in Turkey. *Sci. Am.* **222**, 50–57 (1970).

42. O. Bar-Yosef, "From sedentary foragers to village hierarchies: The emergence of social institutions" in *The origin of human social institutions*, W. G. Runciman, Ed. (Published for the British Academy by Oxford University Press, 2001), vol. 110 of *Proceedings of the British Academy*.

43. K. V. Flannery, The origins of the village revisited: From nuclear to extended households. *Am. Antiq.* **67**, 417–433 (2002).

44. K. Hanghøj, I. Moltke, P. A. Andersen, A. Manica, T. S. Korneliussen, Fast and accurate relatedness estimation from high-throughput sequencing data in the presence of inbreeding. *Gigascience* **8**, 1–9 (2019).

45. J. M. M. Kuhn, M. Jakobsson, T. Günther, Estimating genetic kin relationships in prehistoric populations. *PLOS ONE* **13**, e0195491 (2018).

46. A. Žegarac, L. Winkelbach, J. Blöcher, Y. Diekmann, M. Krečković Gavrilović, M. Porčić, B. Stojković, L. Milašinović, M. Schreiber, D. Wegmann, K. R. Veeramah, S. Stefanović, J. Burger, Ancient genomes provide insights into family structure and the heredity of social status in the early Bronze Age of southeastern Europe. *Sci. Rep.* **11**, 10072 (2021).

47. D. M. Fernandes, O. Cheronet, P. Gelabert, R. Pinhasi, TKGWV2: An ancient DNA relatedness pipeline for ultra-low coverage whole genome shotgun data. *Sci. Rep.* **11**, 1–9 (2021).

48. M. Chyleński, E. Ehler, M. Somel, R. Yaka, M. Krzewińska, M. Dabert, A. Juras, A. Marciniak, Ancient mitochondrial genomes reveal the absence of maternal kinship in the burials of Çatalhöyük people and their genetic affinities. *Genes* **10**, 207 (2019).

49. M. A. Pilloud, C. S. Larsen, "Official" and "practical" kin: Inferring social and community structure from dental phenotype at Neolithic Çatalhöyük, Turkey. *Am. J. Phys. Anthropol.* **145**, 519–530 (2011).

50. C. Meiklejohn, A. Agelarakis, P. M. M. G. Akkermans, P. E. L. Smith, R. Solecki, Artificial cranial deformation in the Proto-neolithic and Neolithic Near East and its possible origin: Evidence from four sites. *Paléorient* **18**, 83–97 (1992).

51. M. Özbek, in *Light on Top of the Black Hill. Studies Presented to Halet Çambel*, G. Arsebük, M. J. Mellink, W. Schirmer, Eds. (Ege Yayınları, 1998), pp. 567–579.

52. M. Özbek, in *Faces from the Past: Diachronic Patterns in the Biology of Human Populations from the Eastern Mediterranean*, M. Fearman, L. K. Horwitz, T. Kahana, U. Zilberman, Eds. (BAR International Series, 2007), pp. 159–162.

53. L. Manouvrier, Incisions, cautérisations et trépanations crâniennes de l'époque néolithique. *Bull. Mém. Soc. Anthropol. Paris* **5**, 67–73 (1904).

54. Y. S. Erdal, Ö. D. Erdal, A review of trepanations in Anatolia with new cases. *Int. J. Osteoarchaeol.* **21**, 505–534 (2011).

55. Y. Erdal, B. Jalilov, M. Koruyucu, V. D'amico, Ö. Erdal, in *Constructing Kurgans. Burial Mounds and Funerary Customs in the Caucasus and Eastern Anatolia During the Bronze and Iron Age, Studies on the Ancient Near East and the Mediterranean*, N. Laneri, G. Palumbi, S. M. Celka, Eds. (Arbor Sapientiae Editore, 2019), pp. 41–55.

56. T. Molleson, S. Campbell, A. Green, Deformed skulls at tell Arpachiyah: The social context, in *The Archaeology of Death in the Ancient Near East* (Oxbow Books, 1995), pp. 45–55.

57. B. Arensburg, I. Hershkovitz, Nahal Hemar cave: Neolithic human remains. *Atiqot.* **18**, 50–58 (1988).

58. P. de Barros Damgaard, R. Martiniano, J. Kamm, J. V. Moreno-Mayar, G. Kroonen, M. Peyrot, G. Barjamovic, S. Rasmussen, C. Zacho, N. Baimukhanov, V. Zaibert, V. Merz, A. Biddanda, I. Merz, V. Loman, V. Evdokimov, E. Usmanova, B. Hemphill, A. Seguin-Orlando, F. E. Yediay, I. Ullah, K.-G. K.-G. Sjögren, K. H. Iversen, J. Choin, C. de la Fuente, M. Ilardo, H. Schroeder, V. Moiseyev, A. Gromov, A. Polyakov, S. Omura, S. Y. Senyurt, H. Ahmad, C. McKenzie, A. Margaryan, A. Hameed, A. Samad, N. Gul, M. H. Khokhar, O. I. Goriunova, V. I. Bazaliiskii, J. Novembre, A. W. Weber, L. Orlando, M. E. Allentoft, R. Nielsen, K. Kristiansen, M. Sikora, A. K. Outram, R. Durbin, E. Willerslev, The first horse herders and the impact of early Bronze Age steppe expansions into Asia. *Science* **360**, eaar7711 (2018).

59. D. Kazancı, N. E. Altınışık, A. Aydoğan, F. Özer, E. Sürer, Ç. Atakuman, M. Somel, Y. Erdal, in *The Archaeology of Anatolia, Volume IV: Recent Discoveries (2018–2020)* (Cambridge Scholars Publishing, 2021), pp. 355–371.

60. T. Carter, A true gift of mother earth: The use and significance of obsidian at Çatalhöyük. *Anatol. Stud.* **61**, 1–19 (2011).

61. T. Carter, S. Dubernet, R. King, F. X. le Bourdonnec, M. Milić, G. Poupeau, M. S. Shackley, Eastern Anatolian obsidians at Çatalhöyük and the reconfiguration of regional interaction in the Early Ceramic Neolithic. *Antiquity* **82**, 900–909 (2008).

62. J. Conolly, *The Çatalhöyük Flint and Obsidian Industry: Technology and Typology in Context* (Archaeopress, 1999), BAR International Series.

63. R. J. Braidwood, H. Çambel, W. Schirmer, Beginnings of village-farming communities in southeastern turkey: Cayönü tepesi, 1978 and 1979. *J. Field Archaeol.* **8**, 249 (1981).

64. K. Schmidt, in *The Neolithic in Turkey: New Excavations & New Research*, M. Özdoğan, N. Başgelen, P. Kuniholm, Eds. (Archaeology & Art Publications, 2011), vol. 2, pp. 41–83.

65. N. Karul, Buried buildings at pre-pottery neolithic Karahantepe. *Türk Arkeoloji ve Etnografya Dergisi.* **82**, 21–31 (2021).

66. P. J. Reimer, E. Bard, A. Bayliss, J. W. Beck, P. G. Blackwell, C. B. Ramsey, C. E. Buck, H. Cheng, R. L. Edwards, M. Friedrich, P. M. Grootes, T. P. Guilderson, H. Haflidason, I. Hajdas, C. Hatté, T. J. Heaton, D. L. Hoffmann, A. G. Hogg, K. A. Hughen, K. F. Kaiser, B. Kromer, S. W. Manning, M. Niu, R. W. Reimer, D. A. Richards, E. M. Scott, J. R. Southon, R. A. Staff, C. S. M. Turney, J. van der Plicht, IntCal13 and Marine13 radiocarbon age calibration curves 0–50,000 years cal BP. *Radiocarbon* **55**, 1869–1887 (2013).

67. C. B. Ramsey, Bayesian analysis of radiocarbon dates. *Radiocarbon* **51**, 337–360 (2009).

68. R. Pinhasi, D. Fernandes, K. Sirak, M. Novak, S. Connell, S. Alpaslan-Roodenberg, F. Gerritsen, V. Moiseyev, A. Gromov, P. Raczky, A. Anders, M. Pietrusewsky, G. Rollefson, M. Jovanovic, H. Trinhhoang, G. Bar-Oz, M. Oxenham, H. Matsumura, M. Hofreiter, Optimal ancient DNA yields from the inner ear part of the human petrous bone. *PLOS ONE* **10**, e0129102 (2015).

69. J. Dabney, M. Knapp, I. Glocke, M. T. Gansauge, A. Weihmann, B. Nickel, C. Valdiosera, N. García, S. Pääbo, J. L. Arsuaga, M. Meyer, Complete mitochondrial genome sequence of a Middle Pleistocene cave bear reconstructed from ultrashort DNA fragments. *Proc. Natl. Acad. Sci. U.S.A.* **110**, 15758–15763 (2013).

70. M. Kircher, S. Sawyer, M. Meyer, Double indexing overcomes inaccuracies in multiplex sequencing on the Illumina platform. *Nucleic Acids Res.* **40**, e3 (2012).

71. M. Schubert, S. Lindgreen, L. Orlando, AdapterRemoval v2: Rapid adapter trimming, identification, and read merging. *BMC. Res. Notes* **9**, 88 (2016).

72. H. Li, R. Durbin, Fast and accurate short read alignment with Burrows–Wheeler transform. *Bioinformatics* **25**, 1754–1760 (2009).

73. M. Kircher, Analysis of high-throughput ancient DNA sequencing data. *Methods Mol. Biol.* **840**, 197–228 (2012).

74. G. Jun, M. K. Wing, G. R. Abecasis, H. M. Kang, An efficient and scalable analysis framework for variant extraction and refinement from population-scale DNA sequence data. *Genome Res.* **25**, 918–925 (2015).

75. P. Skoglund, B. H. Northoff, M. V. Shunkov, A. P. Derevianko, S. Pääbo, J. Krause, M. Jakobsson, Separating endogenous ancient DNA from modern day contamination in a Siberian Neandertal. *Proc. Natl. Acad. Sci. U.S.A.* **111**, 2229–2234 (2014).

76. Q. Fu, A. Mittnik, P. L. F. Johnson, K. Bos, M. Lari, R. Bollongino, C. Sun, L. Giemsch, R. Schmitz, J. Burger, A. M. Ronchitelli, F. Martini, R. G. Cremonesi, J. Svoboda, P. Bauer, D. Caramelli, S. Castellano, D. Reich, S. Pääbo, J. Krause, A revised timescale for human evolution based on ancient mitochondrial genomes. *Curr. Biol.* **23**, 553 (2013).

77. G. Renaud, V. Slon, A. T. Duggan, J. Kelso, Schmutzi: Estimation of contamination and endogenous mitochondrial consensus calling for ancient DNA. *Genome Biol.* **16**, 1–18 (2015).

78. T. S. Korneliussen, A. Albrechtsen, R. Nielsen, ANGSD: Analysis of next generation sequencing data. *BMC Bioinformatics* **15**, 1–13 (2014).

79. P. Skoglund, J. Storå, A. Götherström, M. Jakobsson, Accurate sex identification of ancient human remains using DNA shotgun sequencing. *J. Archaeol. Sci.* **40**, 4477–4482 (2013).

80. R. Martiniano, B. de Sanctis, P. Hallast, R. Durbin, Placing ancient DNA sequences into reference phylogenies. *Mol. Biol. Evol.* **39**, msac017 (2022).

81. I. Letunic, P. Bork, Interactive tree of life (iTOL) v5: An online tool for phylogenetic tree display and annotation. *Nucleic Acids Res.* **49**, W293–W296 (2021).

82. A. Kloss-Brandstätter, D. Pacher, S. Schönherr, H. Weissensteiner, R. Binna, G. Specht, F. Kronenberg, HaploGrep: A fast and reliable algorithm for automatic classification of mitochondrial DNA haplogroups. *Hum. Mutat.* **32**, 25–32 (2011).

83. H. Li, B. Handsaker, A. Wysoker, T. Fennell, J. Ruan, N. Homer, G. Marth, G. Abecasis, R. Durbin, The sequence alignment/map format and SAMtools. *Bioinformatics* **25**, 2078–2079 (2009).

84. S. Mallick, H. Li, M. Lipson, I. Mathieson, M. Gymrek, F. Racimo, M. Zhao, N. Chennagiri, S. Nordenfelt, A. Tandon, P. Skoglund, I. Lazaridis, S. Sankararaman, Q. Fu, N. Rohland, G. Renaud, Y. Erlich, T. Willems, C. Gallo, J. P. Spence, Y. S. Song, G. Poletti, F. Balloux, G. Van Driem, P. De Knijff, I. G. Romero, A. R. Jha, D. M. Behar, C. M. Bravi, C. Capelli, T. Hervig, A. Moreno-Estrada, O. L. Posukh, E. Balanovska, O. Balanovsky, S. Karachanak-Yankova, H. Sahakyan, D. Toncheva, L. Yepiskoposyan, C. Tyler-Smith, Y. Xue, M. S. Abdullah, A. Ruiz-Linares, C. M. Beall, A. Di Rienzo, C. Jeong, E. B. Starikovskaya, E. Metspalu, J. Parik, R. Villems, B. M. Henn, U. Hodoglugil, R. Mahley, A. Sajantila, G. Stamatoyannopoulos, J. T. S. Wee, R. Khusainova, E. Khusnutdinova, S. Litvinov, G. Ayodo, D. Comas, M. F. Hammer, T. Kivisild, W. Klitz, C. A. Winkler, D. Labuda, M. Bamshad, L. B. Jorde, S. A. Tishkoff, W. S. Watkins, M. Metspalu, S. Dryomov, R. Sukernik, L. Singh, K. Thangaraj, S. Paäbo, J. Kelso, N. Patterson, D. Reich, The simons genome diversity project: 300 genomes from 142 diverse populations. *Nature* **538**, 201–206 (2016).

85. M. D. Vigeland, *Pedigree Analysis in R* (Academic Press, 2021).

86. Y. Benjamini, Y. Hochberg, Controlling the false discovery rate: A practical and powerful approach to multiple testing. *J. R. Stat. Soc. Ser. B (Methodological)* **57**, 289–300 (1995).

87. C. F. F. Karney, Algorithms for geodesics. *J. Geodyn.* **87**, 43–55 (2012).

88. 1000 Genomes Project Consortium, A. Auton, L. D. Brooks, R. M. Durbin, E. P. Garrison, H. M. Kang, J. O. Korbel, J. L. Marchini, S. M. Carthy, G. A. McVean, G. R. Abecasis, A global reference for human genetic variation. *Nature* **526**, 68–74 (2015).

89. A. Bergström, S. A. McCarthy, R. Hui, M. A. Almarri, Q. Ayub, P. Danecek, Y. Chen, S. Felkel, P. Hallast, J. Kamm, H. Blanché, J. F. Deleuze, H. Cann, S. Mallick, D. Reich, M. S. Sandhu, P. Skoglund, A. Scally, Y. Xue, R. Durbin, C. Tyler-Smith, Insights into human genetic variation and population history from 929 diverse genomes. *Science* **367**, eaay5012 (2020)

90. N. Patterson, A. L. Price, D. Reich, Population structure and eigenanalysis. *PLOS Genet.* **2**, e190 (2006).

91. D. H. Alexander, J. Novembre, K. Lange, Fast model-based estimation of ancestry in unrelated individuals. *Genome Res.* **19**, 1655 (2009).

92. F. Clemente, M. Unterländer, O. Dolgova, C. E. G. Amorim, F. Coroado-Santos, S. Neuenschwander, E. Ganiatsou, D. I. Cruz Dávalos, L. Anchieri, F. Michaud, L. Winkelbach, J. Blöcher, Y. O. A. Cárdenas, B. S. da Mota, E. Kalliga, A. Souleles, I. Kontopoulos, G. Karamitrou-Mentessidi, O. Philaniotou, A. Sampson, D. Theodorou, M. Tsipopoulou, I. Akamatis, P. Halstead, K. Kotsakis, D. Urem-Kotsou, D. Panagiotopoulos, C. Ziota, S. Triantaphyllou, O. Delaneau, J. D. Jensen, J. V. Moreno-Mayar, J. Burger, V. C. Sousa, O. Lao, A. S. Malaspinas, C. Papageorgopoulou, The genomic history of the Aegean palatial civilizations. *Cell* **184**, 2565–2586.e21 (2021).

93. M. Jakobsson, N. A. Rosenberg, CLUMPP: A cluster matching and permutation program for dealing with label switching and multimodality in analysis of population structure. *Bioinformatics* **23**, 1801–1806 (2007).

94. É. Harney, N. Patterson, D. Reich, J. Wakeley, Assessing the performance of qpAdm: A statistical tool for studying population admixture. *Genetics* **217**, iyaa045 (2021).

95. E. R. Crema, A. Bevan, Inference from large sets of radiocarbon dates: Software and methods. *Radiocarbon* **63**, 23–39 (2021).

96. J. Haslett, A. Parnell, A simple monotone process with application to radiocarbon-dated depth chronologies. *J. R. Stat. Soc. Ser. C* **57**, 399–418 (2008).

97. R Core Team, R: A Language and Environment for Statistical Computing (2021); www.R-project.org/.

98. R. A. M. Villanueva, Z. J. Chen, ggplot2: Elegant graphics for data analysis (2nd ed.). *Interdiscip. Res. Perspect.* **17**, 160–167 (2019).

99. A. Kassambara, ggpubr: “ggplot2” Based Publication Ready Plots (2020); https://CRAN.R-project.org/package=ggpubr.

100. H. Wickham, M. Averick, J. Bryan, W. Chang, L. D. McGowan, R. François, G. Grolemund, A. Hayes, L. Henry, J. Hester, M. Kuhn, T. L. Pedersen, E. Miller, S. M. Bache, K. Müller, J. Ooms, D. Robinson, D. P. Seidel, V. Spinu, K. Takahashi, D. Vaughan, C. Wilke, K. Woo, H. Yutani, Welcome to the tidyverse. *J. Open Source softw.* **4**, 1686 (2019).

101. T. L. Pedersen, patchwork: The composer of plots (2020); https://CRAN.R-project.org/package=patchwork.

102. H. Wickham, Reshaping data with the reshape package. *J. Stat. Softw.* **21**, 1–20 (2007).

103. G. Yu, ggplotify: Convert plot to “grob” or “ggplot” Object (2021); https://CRAN.R-project.org/package=ggplotify.

104. K. Slowikowski, ggrepel: Automatically position non-overlapping text labels with “ggplot2” (2021); https://CRAN.R-project.org/package=ggrepel.

105. G. Yu, emojifont: Emoji and font awesome in graphics (2021); https://CRAN.R-project.org/package=emojifont.

106. T. L. Pedersen, ggforce: Accelerating “ggplot2” (2021); https://CRAN.R-project.org/package=ggforce.

107. R. Bivand, T. Keitt, B. Rowlingson, rgdal: Bindings for the “Geospatial” Data Abstraction Library (2021); https://CRAN.R-project.org/package=rgdal.

108. R. J. Hijmans, raster: Geographic data analysis and modeling (2021); https://CRAN.R-project.org/package=raster.

109. H. Wickham, The split-apply-combine strategy for data analysis. *J. Stat. Softw.* **40**, 1–29 (2011).

110. B. R. Mills, MetBrewer: Color palettes inspired by works at the Metropolitan Museum of Art (2022); https://CRAN.R-project.org/package=MetBrewer.

111. M. D. Vigeland, T. Egeland, pedsuite: Easy installation of the "ped suite" Packages for pedigree analysis (2022); https://CRAN.R-project.org/package=pedsuite.

112. M. Özdoğan, A. Özdoğan, Çayönü: A conspectus of recent work *Paléorient*, 65–74 (1989).

113. D. Baird, A. Fairbairn, E. Jenkins, L. Martin, C. Middleton, J. Pearson, E. Asouti, Y. Edwards, C. Kabukcu, G. Mustafaoglu, N. Russell, O. Bar-Yosef, G. Jacobsen, X. Wu, A. Baker, S. Elliott, Agricultural origins on the Anatolian plateau. *Proc. Natl. Acad. Sci. U.S.A.* **115**, E3077–E3086 (2018).

114. B. L. Peasnall, "The round house horizon along the Taurus-Zagros arc: A synthesis of recent excavations of late Epipaleolithic and early aceramic sites in southeastern Anatolia and northern Iraq," thesis, University of Pennsylvania (2000).

115. T. Watkins, Pushing back the frontiers of mesopotamian prehistory. *Biblical. Archaeol.* **55**, 176–181 (1992).

116. O. Dietrich, J. Notroff, K. Schmidt, in *Feast, Famine or Fighting?* (Springer, 2017), pp. 91–132.

117. D. Stordeur, G. der Aprahmian, M. Brenet, J. C. Roux, Les bâtiments communautaires de Jerf el Ahmar et Mureybet horizon PPNA (Syrie). *Paléorient* **26**, 29–44 (2000).

118. T. Yartah, Tell 'Abr 3, un village du néolithique précéramique (PPNA) sur le Moyen Euphrate. Première approche. *Paléorient.* **30**, 141–158 (2004).

119. I. Caneva, A.-M. Conti, C. Lemorini, D. Zampetti, The lithic production at Çayönü: A preliminary overview of the aceramic sequence. *Neolithic Chipped Stone Industries of the Fertile Crescent.* **1**, 253–266 (1994).

120. I. Caneva, C. Lemorini, Z. Daniela, Lithic technology and functionality through time and space at Çayönü, in *Neolithic Chipped Stone Industries of the Fertile Crescent, and Their Contemporaries in Adjacent Regions* (Ex oriente, 1996), pp. 385–402.

121. S. K. Kozlowski, O. Aurenche, Territories, boundaries and cultures in the Neolithic Near East. *Territories, Boundaries and Cultures in the Neolithic Near East. Année* **32**, 154–156 (2005).

122. S. K. Kozlowski, The gods from Nemrik. *al-Rāfidān* **18**, (1997).

123. M. Özdoğan, N. Başgelen, P. I. Kuniholm, The neolithic in Turkey: New excavations & new research. *Archeol. Art Public.* **1**, 89–127 (2011).

124. S. Jammo, Burying the dead and keeping the living close: Burials and spatial location in the Epi-Paleolithic and Neolithic of the Levant. *ORIENT.* **57**, 93–112 (2022).

125. M. Özdoğan, Transition from the Round Plan to Rectangular-Reconsidering the Evidence of Çayönü, in *Neolithic and Chalcolithic Archaeology in Eurasia: Building Techniques and Spatial Organisation* (Archaeopress, 2010), pp. 29–34.

126. D. Stordeur, F. Abbés, Du PPNA au PPNB: Mise en lumière d'une phase de transition à Jerf el Ahmar (Syrie). *Bulletin de la Société préhistorique française* **99**, 563–595 (2002).

127. M. Białowarczuk, From circle to rectangle. Evolution of the architectural plan in the early Neolithic in the Near East. *Polish Archaeol. Mediterr.* **25**, 575–593 (2016).

128. K. M. Kenyon, *The Architecture and Stratigraphy of the Tell* (British School of Archaeology, 1981), vol. 3.

129. G. Duru, M. Özbaşaran, S. Yelözer, M. Uzdurum, I. Kuijt, Space making and home making in the world's first villages: Reconsidering the circular to rectangular architectural transition in the Central Anatolian Neolithic. *J. Anthropol. Archaeol.* **64**, 101357 (2021).

130. Ç. Atakuman, Architectural discourse and social transformation during the early neolithic of Southeast Anatolia. *J. World Prehist.* **27**, 1–42 (2014).

131. E. Kodaş, Communal architecture at Boncuklu Tarla, Mardin province, Turkey. *Near East. Archaeol.*. **84**, 159–165 (2021).

132. J. J. Shea, *Stone Tools in the Paleolithic and Neolithic Near East: A Guide* (Cambridge Univ. Press, 2015).

133. D. Binder, PPN pressure technology: Views from Anatolia, in *Systemes techniques et communautés du Néolithique précéramique au Proche-Orient (Technical Systems and Near Eastern PPN Communities)* (APDCA, 2007), pp. 235–244.

134. C. Altınbilek-Algül, L. Astruc, D. Binder, J. Pelegrin, "Pressure Blade Production with a Lever in the Early and Late Neolithic of the Near East" in *The Emergence of Pressure Blade Making*, P. M. Desrosiers, Ed. (Springer US, Boston, MA, 2012), pp. 157–179.

135. F. Borrell, La technologie de débitage laminaire bipolaire au Proche-Orient durant le Néolithique précéramique B (PPNB). *J. Lithic Stud.* **4**, 129–161 (2017).

136. D. Binder, Technologie lithique et comportement social dans le PPN de Çayönü tepesi (Turquie). *Un aperçu à travers l'analyse des matières premières. Paléorient.* **34**, 5–21 (2008).

137. J. J. Ibáñez, D. Ortega, D. Campos, L. Khalidi, V. Méndez, L. Teira, Developing a complex network model of obsidian exchange in the Neolithic Near East: Linear regressions, ethnographic models and archaeological data. *Paléorient* **42**, 9–32 (2016).

138. M. L. Antonio, Z. Gao, H. M. Moots, M. Lucci, F. Candilio, S. Sawyer, V. Oberreiter, D. Calderon, K. Devitofranceschi, R. C. Aikens, S. Aneli, F. Bartoli, A. Bedini, O. Cheronet, D. J. Cotter, D. M. Fernandes, G. Gasperetti, R. Grifoni, A. Guidi, F. la Pastina, E. Loreti, D. Manacorda, G. Matullo, S. Morretta, A. Nava, V. F. Nicolai, F. Nomi, C. Pavolini, M. Pentiricci, P. Pergola, M. Piranomonte, R. Schmidt, G. Spinola, A. Sperduti, M. Rubini, L. Bondioli, A. Coppa, R. Pinhasi, J. K. Pritchard, Ancient Rome: A genetic crossroads of Europe and the Mediterranean. *Science* **366**, 708–714 (2019).
